# Characterization of Maize miRNAs in Response to Synergistic Infection of Maize Chlorotic Mottle Virus and Sugarcane Mosaic Virus

**DOI:** 10.3390/ijms20133146

**Published:** 2019-06-27

**Authors:** Zihao Xia, Zhenxing Zhao, Xinran Gao, Zhiyuan Jiao, Yuanhua Wu, Tao Zhou, Zaifeng Fan

**Affiliations:** 1 College of Plant Protection, Shenyang Agricultural University, Shenyang 110866, China; 2 State Key Laboratory of Agro-Biotechnology and Key Laboratory of Pest Monitoring and Green Management-MOA, China Agricultural University, Beijing 100193, China

**Keywords:** maize, miRNA, maize chlorotic mottle virus (MCMV), sugarcane mosaic virus (SCMV)

## Abstract

The synergistic infection of maize chlorotic mottle virus (MCMV) and sugarcane mosaic virus (SCMV) causes maize lethal necrosis, with considerable losses to global maize production. microRNAs (miRNAs) are conserved non-coding small RNAs that play essential regulatory roles in plant development and environmental stress responses, including virus infection. However, the characterization of maize miRNAs in response to synergistic infection of MCMV and SCMV remains largely unknown. In this study, the profiles of small RNAs from MCMV and SCMV single- and co-infected (S + M) maize plants were obtained by high-throughput sequencing. A total of 173 known miRNAs, belonging to 26 miRNA families, and 49 novel miRNAs were profiled. The expression patterns of most miRNAs in S + M-infected maize plants were similar to that in SCMV-infected maize plants, probably due to the existence of RNA silencing suppressor HC-Pro. Northern blotting and quantitative real-time PCR were performed to validate the accumulation of miRNAs and their targets in different experimental treatments, respectively. The down-regulation of miR159, miR393, and miR394 might be involved in antiviral defense to synergistic infection. These results provide novel insights into the regulatory networks of miRNAs in maize plants in response to the synergistic infection of MCMV and SCMV.

## 1. Introduction

RNA silencing is a conserved mechanism in most eukaryotic organisms that regulates the expression of endogenous genes and counteracts viral infections, which is involved in the production of two major groups of small RNAs: microRNAs (miRNAs) and small interfering RNAs (siRNAs) in plants [1,2,3]. The miRNAs are a category of endogenous non-coding small RNAs, typically 21 nt in length, playing essential regulatory roles in plant development and environmental stress responses [1,3]. The gene that encodes miRNA is transcribed to produce primary miRNA (pri-miRNA) by RNA polymerase II (Pol II), which is processed by Dicer-like1 (DCL1) to generate miRNA precursor (pre-miRNA) that contains miRNA/miRNA* sequences with a stem-loop structure [1,4]. The pre-miRNA is further processed by DCL1 to give a miRNA-miRNA* duplex with 2 nt 3′ overhangs that is transported from nucleus into cytoplasm [5]. The mature miRNA binds to an ARGONAUTE (AGO) protein to form RNA-induced silencing complex (RISC), and the miRNA* is usually degraded [3,6]. miRNA directs cleavage or translational inhibition of target mRNA in a sequence-specific manner at the transcriptional and post-transcriptional levels [7,8].

In plants, virus infection triggers RNA silencing and interferes with endogenous miRNA pathways, mostly by virus-encoded RNA silencing suppressor, which can affect the development of disease symptoms [2,9,10,11,12]. For example, the expressions of miR156, miR164, miR165, and miR171 were significantly induced by tobacco mosaic virus (TMV) infection, and their levels were correlated with the severity of symptom in *Nicotiana tabacum* [13]. Additionally, a number of miRNAs have been demonstrated to function in antiviral responses through regulation of their target genes, including important components of RNA silencing, resistance genes, and hormone-associated genes [14,15,16]. Up-regulated miR168 by several plant virus infections mediated the host antiviral defense response by targeting AGO1 [17,18,19]. A monocot-specific miR444 regulated the resistance against rice stripe virus (RSV) infection by up-regulation of *OsRDR1* expression in rice [20]. In tomato, DCL2-dependent miR6026 weakened resistance against potato virus X (PVX) and TMV by targeting *DCL2* mRNAs in a feed-back loop [21]. Bra-miR1885 was induced specifically by turnip mosaic virus (TuMV) infection and targeted a Toll/interleukin receptor (TIR) domain-containing nucleotide-binding site (NBS) leucine-rich repeat (LRR)-class disease-resistant transcript for cleavage in *Brassica rapa* [22]. Moreover, miR6019 and miR6020 guided cleavage of transcripts of the *N* gene that confers resistance to TMV in tobacco [23]. Rice miR319 suppressed jasmonic acid (JA)-mediated defense to facilitate rice ragged stunt virus (RRSV) infection [24]. In maize, it has been reported that miRNAs were differentially regulated by the infection with sugarcane mosaic virus (SCMV) and rice black streaked dwarf virus (RBSDV), respectively [25,26,27].

Maize chlorotic mottle virus (MCMV), the only member of the genus *Machlomovirus* in the family *Tombusviridae*, can infect various crops and lead to typical mild mosaic symptoms [28]. When maize plants are co-infected with MCMV and SCMV, maize dwarf mosaic virus (MDMV), or wheat streak mosaic virus (WSMV), leaves and stems of infected plants exhibit a severe systemic necrosis known as maize lethal necrosis (MLN), which causes considerable yield losses in maize production worldwide [28,29,30]. In China, MCMV was first observed in maize with necrotic and chlorotic leaves and stems in 2009, and the observed MLN disease was caused by synergistic infection with MCMV and SCMV [31,32]. Our previous study has demonstrated that synergistic infection of MCMV and SCMV increased the accumulations of both MCMV and MCMV-derived siRNAs in maize [18]. However, the effects of synergistic infection of MCMV and SCMV on maize endogenous miRNA pathways have not been elucidated. In this study, we used small RNA sequencing to investigate the characterization of maize miRNAs responsive to synergistic infection of MCMV and SCMV at 9 days post inoculation (dpi). Moreover, Northern blotting and quantitative real-time reverse transcription-polymerase chain reaction (qRT-PCR) were performed to determine the accumulations of several known and novel miRNAs and their predicted target genes, respectively. These results contribute to understanding the possible roles of miRNAs and their targets in maize plants responsive to MLN.

## 2. Results

### 2.1. High-Throughput Sequencing of Small RNAs

To survive and reproduce in plants, viruses can modulate host cellular functions and resources by interfering with host endogenous miRNA pathways [12]. Our previous research has demonstrated that the first systemically infected leaves of co-infection of MCMV and SCMV became significantly chlorotic at 9 dpi and developed necrotic areas at 10 dpi [18]. To investigate the effects of synergistic infection of MCMV and SCMV on characterization of maize miRNAs, four small RNA profiles were obtained from the systemically infected leaves of maize plants inoculated with buffer (Mock), SCMV, MCMV, and SCMV + MCMV (S + M) at 9 dpi by high-throughput sequencing, respectively (Table 1). A total of 9,759,612, 9,892,919, 10,217,242, and 11,026,769 clean reads, and 2,126,945, 1,259,696, 2,180,375, and 1,253,995 unique reads were obtained from small RNA libraries of Mock-, SCMV-, MCMV-, and S + M-inoculated maize plants, respectively (Table 1). These sequences were then mapped to their precursors of maize miRNAs in miRBase (version 21.0), obtaining 184,429, 323,206, 170,066, and 130,396 total reads and 3981, 5216, 3806, and 3516 unique reads from Mock, SCMV, MCMV and S + M libraries, respectively (Table 1). Moreover, a total of 6,740,592 and 8,565,445 virus-derived siRNAs (vsiRNAs) were obtained, accounting for 68.14% and 77.68% of total clean reads in SCMV- and S + M-inoculated maize plants, respectively (Table 1).

**Figure 1:**
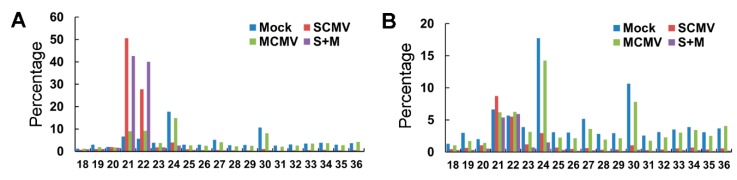
Size distribution of small RNAs from Mock-, SCMV-, MCMV-, and S + M-inoculated maize plants. (**A**) Size distribution of total reads of small RNAs from Mock-, SCMV-, MCMV-, and S + M-inoculated maize plants. (**B**) Size distribution of total reads of small RNAs (without vsiRNAs) from Mock-, SCMV-, MCMV-, and S + M-inoculated maize plants.

To explore the roles of different categories of small RNAs in response to virus infections, the size distribution of small RNAs was analyzed (Figure 1). The results showed that the accumulations of 21- and 22-nt small RNAs increased in both SCMV and S + M infected maize plants (Figure 1A). After removal of vsiRNAs, the increased accumulations of 21- and 22-nt small RNAs from SCMV and S + M library significantly decreased, indicating that the increased small RNAs were mainly composed of vsiRNAs in SCMV- and S + M-inoculated maize plants (Figure 1B). Interestingly, SCMV infection increased the accumulation of 21-nt small RNAs except for vsiRNAs, mainly due to the increased miRNAs (Figure 1B and Table 1). The accumulation of 24-nt small RNAs was decreased in SCMV- and S + M-inoculated maize plants, which was predominant in healthy maize plants (Figure 1), probably because the existence of vsiRNAs affected the proportion of small RNAs.

### 2.2. The Expression of Known miRNAs

**Figure 2:**
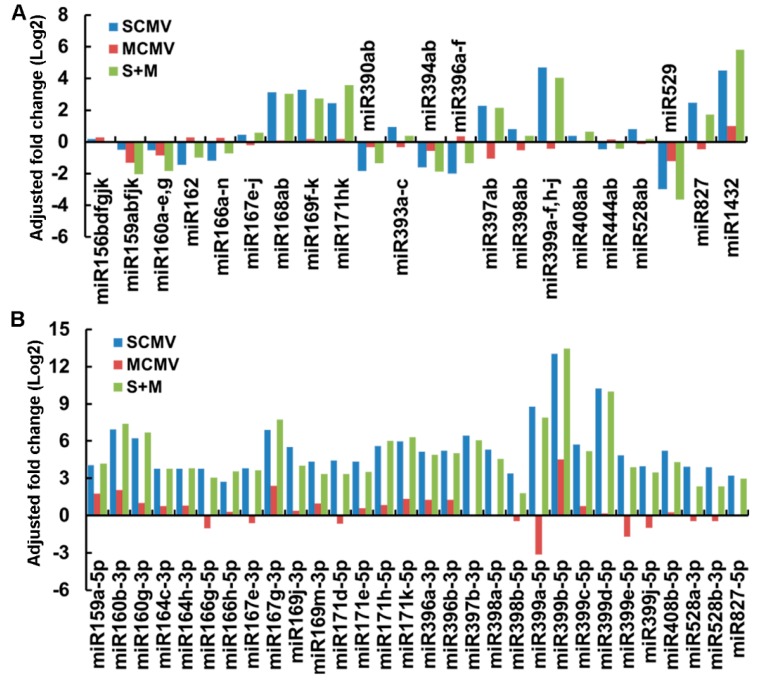
Differential expression of microRNAs (miRNAs) (**A**) and miRNA*s (**B**) in SCMV-, MCMV-, and S + M-infected maize plants.

**Figure 3:**
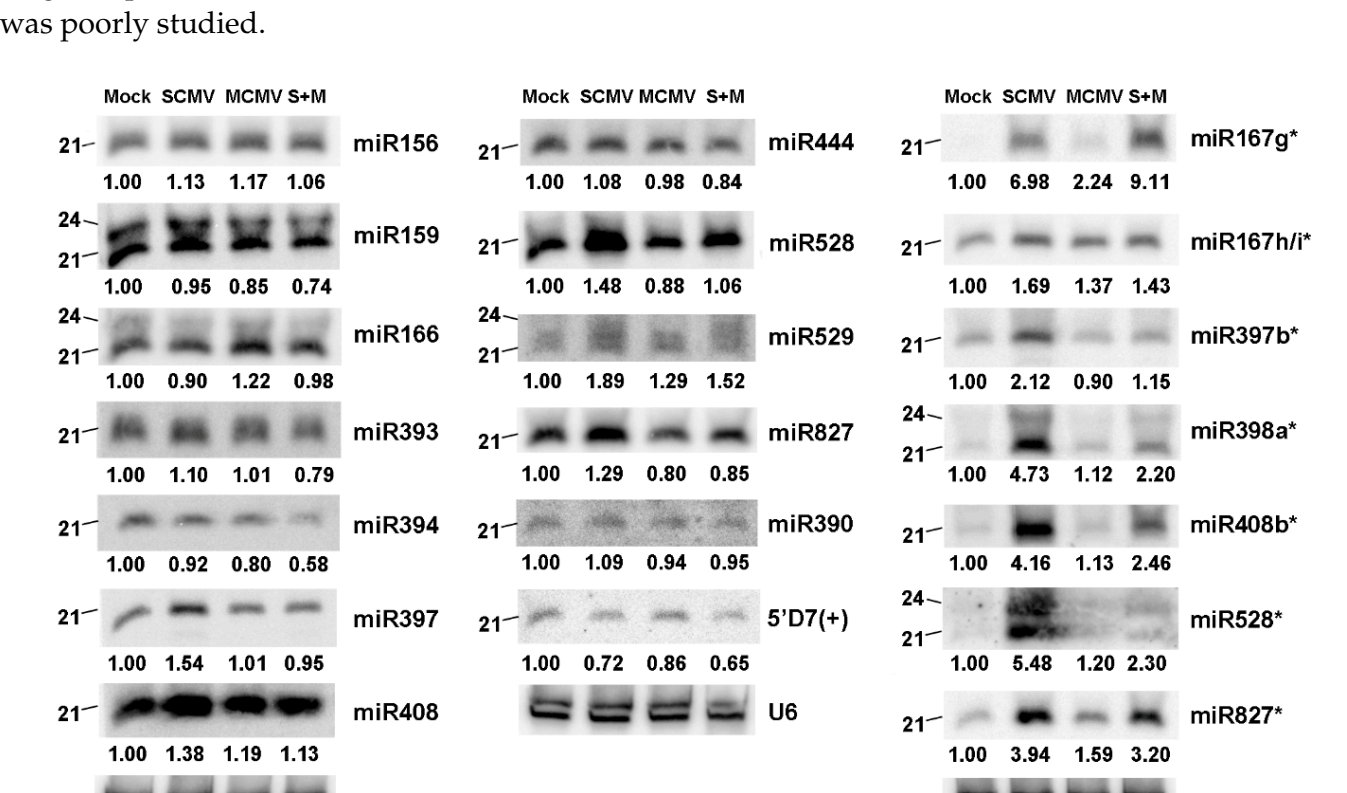
The accumulation of small RNAs in Mock-, SCMV-, MCMV-, and S + M-inoculated maize plants. The accumulation of small RNAs was detected at 9 dpi by Northern blotting. The samples from three independent experiments with at least three biological replicates each were pooled for RNA extraction. U6 was used as a loading control. The expression levels of small RNAs from Mock-inoculated plants (negative controls) were set to a value of 1.00 as reference, and these from SCMV-, MCMV-, or S + M-inoculated maize plants were counted relative to the levels of Mock-inoculated maize plants from the same experiments using the Image J software.

A total of 173 known miRNAs were identified by alignment to miRBase 21.0, belonging to 26 miRNA families (Table S1). To determine the expression levels of these miRNAs in different libraries, the reads were normalized as transcripts per million (TPM) (Table S1). Due to the existence of vsiRNAs that affected the proportion of endogenous small RNAs, the fold change of individual miRNA was normalized to that of miR156a (Figure 2), of which the expression level assayed by Northern blotting was nearly unchanged after virus infections (Figure 3). The results showed that the expression levels of most miRNAs were differentially expressed in SCMV- and S + M-inoculated maize plants, while there was no significant difference in MCMV-inoculated maize plants (Figure 2A). In SCMV and S + M libraries, miR168, miR169, miR171, miR397, miR399, miR827, and miR1423 had >two-fold abundance, while miR159, miR160, miR162, miR166, miR390, miR394, miR396, and miR529 were down-regulated compared with that in Mock-inoculated maize plants (Figure 2A). Moreover, SCMV single and co-infection with MCMV increased the accumulations of all of miRNA*s (Figure 2B). These results revealed that the expression patterns of most miRNAs in S + M-infected maize plants were similar to those in SCMV-infected maize plants (Figure 2).

To confirm the results of high-throughput sequencing, we performed Northern blotting to validate the expression levels of several known miRNAs and miRNA*s in Mock-, SCMV-, MCMV-, and S + M-inoculated maize plants using the pooled samples from other three independent experiments with at least three biological replicates each (Figure 3). The results showed that miR397, miR408, miR528, miR529, and miR827 were up-regulated after SCMV infection, whilst 5′D7(+) was down-regulated (Figure 3). MCMV infection increased the accumulations of miR166 and miR529, and decreased the expressions of miR159, miR394, miR827, and 5′D7(+) (Figure 3). The accumulations of miR159, miR393, miR394, miR444, miR827, and 5′D7(+) were inhibited in S + M-infected maize plants, but only miR529 was up-regulated (Figure 3). Compared to SCMV and MCMV single infection, the accumulations of most miRNAs decreased in S + M-infected maize plants, indicating that the down-regulation of miR159, miR393, and miR394 might be involved in antiviral defense with regard to synergistic infection of MCMV and SCMV. Additionally, the accumulations of selected miRNA* were significantly induced in SCMV- and S + M-inoculated maize plants, whilst only miR167g, h/i*, miR528*, and miR827* were up-regulated after MCMV infection (Figure 3). These results suggested that the data of Northern blotting were mostly consistent with the results of small RNA sequencing. Intriguingly, we found that miRNAs with 24-nt in length were identified in miR159, miR166, miR529 (also 22-nt), miR398a*, and miR528* by Northern blotting, suggesting that the size of these miRNAs was diverse although their length deposited in miRBase database was 21-nt. However, the function of these 22- or 24-nt miRNAs was poorly studied.

### 2.3. The Prediction and Expression of Novel miRNAs

To identify a novel miRNA, possessing certain reads, a characteristic stem-loop precursor and/or a corresponding miRNA* have been strong evidences [33]. In this study, only the candidate miRNAs represented by not less than 3 TPM reads in one of four libraries were identified as novel miRNAs. A total of 49 novel miRNAs were predicted in four libraries, of which 20 were mapped to more than one loci in maize genome (Table 2). Among these novel miRNAs, 15 complementary sequences (miRNA*) were identified, 24 novel miRNAs were 21-nt in length, and U was the most abundant nucleotide at the 5′-terminal (40.82%) (Table 2). The precursors of novel miRNAs varied from 50- to 260-nt, meeting the criterion of limitation precursor length to 300 nucleotides [33]. The minimum folding free energy index (MFEI) ranged from 0.51 to 1.92, and the average MFEI value was 1.11 (Table 2), which was a sufficient criterion to distinguish miRNAs from coding and other non-coding RNAs [34]. The secondary structures of novel miRNA precursors were obtained (Figure S1). Further analysis revealed that 19 novel miRNAs were expressed in all four libraries; miRn-07, miRn-42, and miRn-46 were specifically induced after virus infections; miRn-04 and miRn-22 were specific to SCMV-inoculated maize plants, miRn-39 was specific to MCMV-inoculated maize plants, and miRn-11, miRn-14, miRn-32, miRn-44, and miRn-47 to S + M-inoculated maize plants; miRn-01 and miRn-30 were induced by virus single-infection; seven novel miRNAs were only obtained in both SCMV and S + M libraries (Table 2).

**Figure 4:**
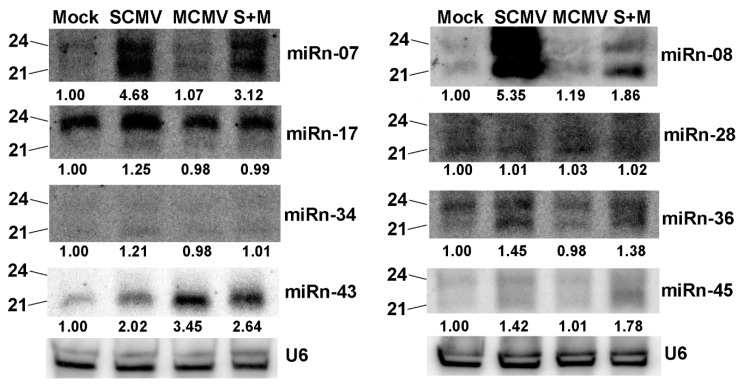
The expression of several novel miRNAs in Mock-, SCMV-, MCMV-, and S + M-inoculated maize plants. The accumulation of small RNAs was detected at 9 dpi by Northern blotting. The samples from three independent experiments with at least three biological replicates each were pooled for RNA extraction. U6 was used as a loading control. The expression levels of small RNAs from Mock-inoculated plants (negative controls) were set to a value of 1.00 as reference, and those from SCMV-, MCMV-, or S + M-inoculated maize plants were counted relative to the levels of Mock-inoculated maize plants from the same experiments using the Image J software.

Because the existence of vsiRNAs affected the proportion of endogenous small RNAs (Table 1 and Figure 1), the normalized reads in the data of high-throughput sequencing could not present the real expression levels of novel miRNAs (Table 2). To confirm the existence and expression of novel miRNAs, Northern blotting assays were performed (Figure 4). The results demonstrated that most miRNAs accumulated as 21- and 24-nt in length, whilst the majority of miRn-17 and miRn-43 were 24-nt and 21-nt, respectively. Although the length of several novel miRNAs was inconsistent with that shown in Table 2, the existence of predicted miRNAs was validated, indicating the method of predicting novel miRNAs was feasible. The length diversity of novel miRNAs was also found in conserved miRNAs (Figure 3), suggesting that these miRNAs might be multifunctional and involved in various regulation pathways. Additionally, miRn-07, miRn-08, miRn-36, and miRn-45 were up-regulated in SCMV- and S + M-inoculated maize plants, and the accumulation of miRn-17 was slightly increased after SCMV infection. Strikingly, the expression of miRn-43 was induced in all experimental treatments of virus infections, especially in MCMV-inoculated maize plants. However, the signal of miRn-34 was weak, and the change of its accumulation could not be counted accurately.

### 2.4. The Prediction and Expression of miRNA Target Genes

It has been demonstrated that the roles of miRNAs in various biological pathways depend on the regulation of their target genes [8]. In our previous report, the target genes of most known miRNAs were obtained by degradome analysis [27]. To explore the roles of novel miRNAs, the miRanda algorithm was applied to predict the target genes of novel miRNAs. Thousands of target genes were obtained, and only those whose scores were not less than 180 were presented (Table S2). Most novel miRNAs were predicted to regulate more than one target gene, whilst 20 novel miRNAs had no predicted target gene within the threshold value. These obtained miRNA targets were involved in a wide range of biological process and played important regulatory roles in plant growth and development.

**Figure 5:**
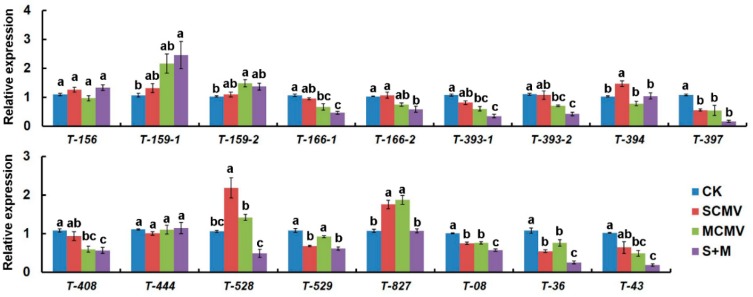
The expression of miRNA target genes in Mock-, SCMV-, MCMV-, and S + M-inoculated maize plants. The expression levels were determined by qRT-PCR at 9 dpi. Three independent experiments were conducted with at least three biological replicates each. Error bars represent the means ± SE. Different letters on the error bars indicate significant differences among samples according to ANOVA followed by Tukey’s HSD multiple comparison (*p*  <  0.05).

To understand the roles of miRNAs involved in the interaction between viruses and maize plants, qRT-PCR was carried out to determine the expression levels of miRNA target genes (Figure 5). The results revealed that SCMV infection induced the accumulations of target genes of miR394 (*T-394*), *T-528,* and *T-827*, while inhibited that of *T-397*, *T-529*, *T-08,* and *T-36*. The expressions of *T-166-1*, *T-393-1*, *T-393-2*, *T-397*, *T-408*, *T-08*, *T-36,* and *T-43* were down-regulated in MCMV-inoculated maize plants, whilst *T-159-2* and *T-827* were up-regulated. In S + M-inoculated maize plants, the accumulations of most target genes were decreased, including *T-166-1*, *T-166-2*, *T-393-1*, *T-393-2*, *T-397*, *T-408*, *T-529*, *T-08*, *T-36,* and *T-43*, yet only *T-159-1* increased.

## 3. Discussion

Maize is a major food crop and an important source of starch and biofuel for industry worldwide, and also a model plant for biological research [35]. As with many other crops, maize production is threatened by pathogens, pests, and abiotic stresses [36]. Over the past ten years, MLN has emerged in sub-Saharan East Africa, Southeast Asia, and South America, with large impacts on maize production [28]. Recently, genome-wide miRNAs involved in plant development and environmental stress responses have been identified by high-throughput sequencing, and the roles of several specific miRNAs have been reported in maize [12]. Although the miRNAs involved in SCMV and RBSDV infection in maize have been elucidated [25,26,27], the characterization of miRNAs in response to synergistic infection of SCMV and MCMV has not been explored. In this study, we obtained 10,042,093, 10,107,781, 10,544,484, and 11,306,497 raw reads by high-throughput sequencing from small RNA libraries of Mock-, SCMV-, MCMV-, and S + M-inoculated maize plants, respectively (Table 1). A total of 173 known miRNAs and 49 novel miRNAs were profiled, of which most were differentially expressed after virus infections (Figure 2). Moreover, amounts of vsiRNAs were obtained in virus-infected libraries, especially in SCMV- and S + M-inoculated maize plants, which had a significant impact on the category and composition of small RNAs (Table 1). Interestingly, we found that the ratio of total miRNA reads in annotated sequences increased (Mock, 4.74%; SCMV, 26.65%; MCMV, 4.91%; and S + M, 16.43%) in SCMV- and S + M-inoculated maize plants (Table 1), suggesting that SCMV single- or co-infection with MCMV induced the accumulation of miRNAs and/or miRNA*s. As a viral suppressor of RNA silencing, potyvirus-encoded P1/HC-Pro has been demonstrated to affect the accumulation of miRNAs and interfere with their function in transgenic plants [9,37]. The differential accumulations of maize miRNAs and/or miRNA*s in SCMV- and S + M-inoculated maize plants might be the results of SCMV HC-Pro accumulation in the process of virus infections. However, the roles of SCMV HC-Pro in interfering with the characterization of maize miRNAs in response to synergistic infection of SCMV and MCMV need to be further investigated.

miRNAs have been known to play very important roles in plant development and environmental stress responses [12]. Although many miRNAs are conserved among different organisms, specific and/or novel miRNAs can be induced in response to biotic and/or abiotic stress responses [16]. Identifying novel miRNA is therefore a critical step to improve the understanding of biological regulation. In this study, 49 novel miRNAs were obtained under certain conditions, of which 19 were induced by virus infections (Table 2). Moreover, the sequences of six novel miRNAs (miRn-02, miRn-17, miRn-24, miRn-28, miRn-30, and miRn-45) were identical to those in our previous report [27]. Interestingly, we found that miRn-12 and miRn-16 belonged to miR156 and miR166 family, respectively (Table 2). These results demonstrated that the method used to predict novel miRNAs was feasible.

It has been reported that the regulatory pathways of plant miRNAs are associated with virus infections, which are involved in viral symptoms and/or pathogenicity by regulating their target genes [12,16]. In this study, a number of miRNAs in maize were identified to be involved in synergistic infection of MCMV and SCMV. In maize plants infected with different viruses, the expression pattern of different miRNAs showed diversification. In the first group, the accumulations of five known (miR166, miR397, miR408, miR528, and miR529) and six novel miRNAs (miRn-07, miRn-08, miRn-17, miRn-34, miRn-43, and miRn-45) increased after virus infections (Figure 4 and Figure 5). The expression of miR166 was induced specifically by MCMV infection, which is involved in the regulation of shoot apical meristem development [38,39]. The accumulations of miR397, miR408, and miR528 increased in SCMV-infected maize plants, of which miR397 plays regulatory roles in rice yield, lignin biosynthesis in wood formation, and symbiotic nitrogen fixation [40,41,42]; miR408 targets copper-containing proteins and responds to various abiotic stresses, *Plantacyanin* regulation, and stripe rust [43,44,45]; and miR528 is involved in multiple stress responses [46,47,48], including RSV infection in rice [49]. After virus infections, miR529 and miRn-43 were up-regulated, of which miR529 is evolutionarily related to miR156 and regulates bract development and the establishment of meristem boundaries in maize [50], and miRn-43 targets a Kinesin-like protein KIN-7K (chloroplast) that is very important for the generation and/or the maintenance of cp-actin filaments involved in chloroplast movement and positioning [51]. The accumulations of miRn-07, miRn-08, miRn-17, miRn-34, and miRn-45 were induced in SCMV- and S + M-inoculated maize plants, while the function of these novel miRNAs needs to be further investigated. In the second class, four miRNAs (miR159, miR393, miR394, and miR444) were down-regulated in response to virus infections (Figure 4). miR159 and miR394 were down-regulated in both MCMV- and S + M-infected maize plants, of which miR159 is associated with disease symptom induction by a severe strain of cucumber mosaic virus in *Arabidopsis* [52], the accumulations of phased siRNAs from miR159 precursors are enhanced after RSV infection in rice [53], and miR394 is involved in response to salt and drought stress and *Fusarium oxysporum* infection [54,55]. The expressions of miR393 and miR444 decreased after MCMV infection, of which miR393 is induced by bacterial infection and negatively regulates auxin signaling [56], and miR444 targets MADS transcription factor to modulate antiviral response [20]. The third class only includes the miR827 that was induced by SCMV infection, whilst decreased by MCMV and S + M infection. miR827 has been reported to control phosphate homeostasis and mediate plant susceptibility to *Heterodera schachtii* [57,58]. These expression patterns of different miRNAs in response to virus infections provide novel insights into the roles of miRNAs in the interaction between virus and host plant. However, the molecular mechanism underlying specific miRNAs involved in synergistic infection of MCMV and SCMV remains to be investigated.

In plants, miRNAs regulate their target genes mainly by mRNA degradation, resulting in an inverse correlation of accumulation between miRNAs and their target mRNAs. However, this inverse relationship cannot be always observed, even in the presence of miRNA-mediated degradation evidence or validated targets, such as miR168 and miR528 [18,27]. It has been reported that the induction of miR168 by virus infections negatively regulates the expression of AGO1 protein, while the accumulation of *AGO1* mRNA is enhanced in parallel as a host defense reaction [17]. Recently, viral-inducible AGO18 has been demonstrated to sequester miR168 and miR528 to alleviate repression of *AGO1* and *L-ascorbate oxidase* mRNAs, respectively, thereby enhancing antiviral defense of rice [19,49]. Moreover, miR528 was shown to be located on polyribosome fractions, suggesting a role for miR528 in regulation of target genes at the level of translation in maize [59]. The induced AGO18 by SCMV, MCMV, and synergistic infection may interfere with the accumulation of target genes of miRNAs that associated with AGO18 [18]. Additionally, the relationship of accumulation between miRNAs and their target genes has been revealed to depend on the tissue or the environmental process [7,59]. Our results highlight the complexity of miRNA-mediated regulatory networks in response to synergistic infection of MCMV and SCMV in maize.

## 4. Materials and Methods

### 4.1. Plant Growth and Virus Inoculations

Maize (*Zea mays* L.) inbred line B73 plants were planted in growth chambers (28 °C day and 22 °C night, 16 h light and 8 h dark cycles) for plant growth and virus inoculation. SCMV-BJ was collected from previously published sources [27]. MCMV was obtained as reported previously [18]. SCMV- or MCMV-infected maize leaf tissues were collected, and the crude extracts were prepared as described previously [18]. The crude extracts were then equally mixed as the source of co-infection, while equal volume of phosphate buffer was added for single infection, respectively.

### 4.2. Small RNA Sequencing and Bioinformatics Analyses

The systemically infected leaves were harvested at 9 dpi and maintained at −80 °C. At least 15 maize seedlings were pooled for small RNA sequencing for each treatment. Total RNA was extracted using TRIzol reagent (Invitrogen, Carlsbad, CA, USA) and subjected to Solexa/Illumina sequencing by SBC (Shanghai Biotechnology Corporation, Shanghai, China).

The clean reads were obtained after excluding low quantity reads and 5′- and 3′-adaptor contaminants, which were then used to query miRBase Database (version 21.0, http://www.mirbase.org) and Rfam Database (version 10, http://rfam.janelia.org). The reads mapped to miRNA precursors by BLAST were identified as known miRNAs. Novel miRNAs were predicted using miRCat of the UEA small RNA workbench as reported previously [27].

### 4.3. Target Gene Prediction

The miRanda algorithm was used to predict target genes of novel miRNAs [60]. Briefly, the criteria used were as follows: (1) no more than four mismatches between miRNA and target (G-U bases count as 0.5 mismatches), (2) no more than two adjacent mismatches in the miRNA/target duplex, (3) no adjacent mismatches in positions 2–12 of the miRNA/target duplex (5′-terminus of miRNA), (4) no mismatches in positions 10–11 of miRNA/target duplex, (5) no more than 2.5 mismatches in positions 1–12 of the miRNA/target duplex (5′-terminus of miRNA), (6) the predicted complementary structure between miRNA and target has a high minimal folding free energy (MFE) that must be no less than 75% of the best complementary structure.

### 4.4. Northern Blotting Analysis

Approximately 40 µg of total RNA was prepared for Northern blotting analysis of known and novel miRNAs. The samples used for qRT-PCR assays were pooled for RNA extraction from other three independent experiments with at least three biological replicates each. The total RNA was separated in a 15% urea polyacrylamide gel, electrophoretically transferred to Hybond-NX membrane (GE Healthcare, Buckinghamshire, UK) using a semi-dry transfer apparatus (Amersham Biosciences, Piscataway, NJ, USA), and was chemically cross-linked via 1-ethyl-3-(3-dimethylaminopropyl) carbodiimide (EDC). For labeling reaction of probes, 1 µL of 10 µM probes, 2.5 µL of 10 × T4 PNK buffer (New England Biolabs, Beverly, MA, USA), 1 µL of [γ-^32^P] ATP (~10 µCi/µL), 19.5 µL of ddH_2_O, and 1 µL of T4 Poly Nucleotide Kinase (New England Biolabs, Beverly, MA, USA) were added (a total volume of 25 µL reaction) and kept in a water bath for 1 h at 37 °C. Blots were pre-hybridized and hybridized at 42 °C overnight using ULTRAhyb^®^-Oligo hybridization buffer (Sigma-Aldrich, St Louis, MO, USA). Post-hybridization washes were performed using 2× SSC and 0.2% sodium dodecyl sulfate (SDS) at 42 °C for 20 min for twice. Hybridization signals were detected by exposing blots to autoradiographic film. The sequences of probes used for Northern blotting analysis were listed (Table S3).

### 4.5. Quantitative Real-time RT-PCR

Total RNA was extracted using TRIzol reagent (Invitrogen, Carlsbad, CA, USA) and treated with DNase I (TaKaRa Bio Inc., Dalian, China). The samples from three independent experiments with at least three biological replicates each were used. About 2 µg of total RNA was reverse-transcribed into first-strand cDNA, and qRT-PCR was performed as reported previously [18]. The sequences of primers used for qRT-PCR are listed in Table S4.

## 5. Conclusions

It has been demonstrated that miRNAs play essential regulatory roles in response to virus infection. The synergistic infection of MCMV and SCMV causes maize lethal necrosis and results in considerable losses to global maize production. However, how maize miRNAs function in response to synergistic infection of MCMV and SCMV is poorly studied. In this study, we obtained four profiles of small RNAs from MCMV and SCMV single- and co-infected maize plants by high-throughput sequencing. A total of 173 known miRNAs were profiled, and 49 novel miRNAs were predicted. Virus infections affected the accumulations of most miRNAs, and the expression patterns of several miRNAs in S + M-inoculated maize plants were similar to that in SCMV-inoculated maize plants. Additionally, SCMV single and synergistic infection induced the accumulations of almost all of miRNA*s. The accumulations of miRNAs and their target genes were determined in buffer-, SCMV-, MCMV-, and S + M-inoculated maize plants by Northern blotting and qRT-PCR assays, respectively. These results provide novel insights into the regulatory networks of maize miRNAs in response to synergistic infection of MCMV and SCMV.

## Figures and Tables

**Table 1:** Small RNAs from buffer (Mock)-, sugarcane mosaic virus (SCMV)-, maize chlorotic mottle virus (MCMV)-, and SCMV + MCMV (S + M)-inoculated maize plants.

Category	Mock		SCMV		MCMV		S+M	
	Total	Unique	Total	Unique	Total	Unique	Total	Unique
Raw reads	10,042,093	-	10,107,781	-	10,544,484	-	11,306,497	-
Clean reads	9,759,612	2,126,945	9,892,919	1,259,696	10,217,242	2,180,375	11,026,769	1,253,995
Annotated sequences ^a^	3,889,735	228,265	1,212,784	114,841	3,466,463	215,610	793,418	86,612
miRBase (version 21.0)	184,429	3981	323,206	5216	170,066	3806	130,396	3516
ncRNA	3,140,721	138,394	731,977	72,289	2,848,339	132,250	549,274	58,696
Rfam (version 10) ^b^	564,585	85,890	157,601	37,336	448,058	79,554	113,748	24,400
Unannotated sequences	5,869,877	1,898,680	8,680,135	1,144,855	6,750,779	1,964,765	10,233,351	1,167,383
SCMV-derived siRNAs ^c^	-	-	6,740,592	-	-	-	6,520,905	-
MCMV-derived siRNAs ^c^	-	-	-	-	1,255,641	-	2,044,540	-

^a^ Encompass the defined small RNA sequence ±2 nt on each side and map within two mismatches; ^b^ Rfam: collection of many common non-coding RNA families except miRNA; http://rfam.janelia.org; ^c^ Refer to [18].

**Table 2:** The characterization of novel miRNAs.

Novel miRNA	Sequence	Size	Reads ^a^				LP	MFEI	miRNA*	Loci
			Mock	SCMV	MCMV	S + M				
miRn-01	UUGUUGUGUUUCAACUCUAGCCU	23	0.00	3.23	18.79	0.00	88	0.66		1
miRn-02	UUAAAUCUGGACCCUUCAUCU	21	8.20	1.82	5.97	1.63	200	1.33	Yes ^b^	1
miRn-03	UGUCGUGCACUUGGUGAACACC	22	5.74	5.16	8.03	3.26	179	0.71		1
miRn-04	UGUCAAUAAGGGCCUGCCUCUGA	23	0.00	5.76	0.00	0.00	174	0.96		1
miRn-05	UGUCAAUAAGGGCCUACCUCUGA	23	2.87	56.10	2.35	7.71	175	0.99		1
miRn-06	UGGCGAUGGAAGCUCUGCUUC	21	0.00	6.27	0.00	1.72	174	0.94		1
miRn-07	UGGCGAUGAGAGUGGUAGCUC	21	0.00	33.86	0.59	9.16	235	0.84	Yes	1
miRn-08	UGCCUGCCUCUUCCAUUCCUUC	22	28.18	16.58	17.81	3.08	145 ^c^	0.90 ^d^	Yes	2
miRn-09	UGCAUUUUUAGGUCCUUGAAC	21	0.00	3.74	0.00	3.63	207	1.08	Yes	1
miRn-10	UGACUCACUCUUACCGCCCAUG	22	0.00	5.66	0.00	2.27	124	0.65		1
miRn-11	UGACGCAACACCGUUGGAUGU	21	0.00	0.00	0.00	5.99	235	1.69	Yes	1
miRn-12	UGACAGAAGAGAGUGAGCACA	21	4.71	12.84	7.05	7.35	177	0.92		7
miRn-13	UGAACUUUUGUACUUUUGGGCC	22	2.66	0.51	3.23	0.00	216	1.57		1
miRn-14	UGAACACCAUGCUGUUGGCUCC	22	0.00	0.00	0.00	4.90	102	0.64		1
miRn-15	UCGGUUUUGUGGCUUCCAAAC	21	0.00	4.04	0.00	0.91	129	1.32	Yes	6
miRn-16	UCGGACCAGGCUUCAUUCCCC	21	18867	3170	21041	2736	150	0.89		7
miRn-17	UCCAGACGUAACCGAACAAGC	21	3.79	3.84	2.94	2.54	145	1.04		7
miRn-18	UCCAAUCUUCCCGUGAUCCCG	21	3.07	1.01	4.01	0.54	120	1.35	Yes	1
miRn-19	UACUUGACUGAGGUGCUUGGCC	22	0.72	0.00	3.03	0.00	190	0.71		1
miRn-20	UAAUUACAUAGGUUAGGACUA	21	2.25	3.03	2.84	1.63	202	1.72		1
miRn-21	GGCCCGCCGAUCACGUCGUGC	21	16.91	7.58	7.24	0.00	107	0.74		2
miRn-22	GAGGAUUGAAGGGAUUAAAUC	21	0.00	3.23	0.00	0.00	107	1.39		1
miRn-23	GAGAGAAUCUGGCUGUGAGAAGA	23	3.38	0.81	1.08	0.00	118	0.70		4
miRn-24	CGGGAACUGGAGAUGCUACUC	21	17.52	4.25	17.42	5.26	203	1.06	Yes	1
miRn-25	CGAGAGUGACGAAGAAAAUCGA	22	1.74	0.00	6.66	0.00	142	0.68		2
miRn-26	CCUUAAUAAUCUGAAUCCGCGG	22	3.89	4.75	0.00	1.90	226	1.51		1
miRn-27	CCGUGGCUCCUGCUCCUGAUG	21	0.00	3.64	0.00	0.73	186	0.98		2
miRn-28	CCCGCCGGCGAGCGCUUUCCU	21	143.55	11.52	154.64	12.06	132	0.83	Yes	4
miRn-29	CACUUUAGGACCACAAAUAAG	21	0.92	4.65	0.00	2.09	153	1.53		6
miRn-30	CACGGAUACUUUUGGGGCACC	21	0.00	11.83	16.44	0.00	110	0.68		1
miRn-31	CACCAACUACUGAUUCAGAGGC	22	1.13	0.00	5.68	2.18	101	0.64		1
miRn-32	CAAGCAUAAAGGUGAAAGGACC	22	0.00	0.00	0.00	3.36	210	1.21	Yes	1
miRn-33	CAAAGAGAAUUGAGGGGGCUA	21	0.00	7.58	0.00	2.18	139	1.61		6
miRn-34	AUUUUAGCCCCUCCAAUCUCC	21	7.27	2.93	10.47	1.90	146	1.28		9
miRn-35	AUUGGAGGGGAUUGAGGAGGCU	22	7.79	4.04	3.91	1.18	143	1.48		2
miRn-36	AUGCGGAGAGGCUCUCGAGAGA	22	47.95	91.38	153.17	52.51	189	0.92	Yes	1
miRn-37	AGUUCGGUCAUCCUGUAGUGAC	22	4.92	1.52	6.26	1.81	201	1.16	Yes	2
miRn-38	AGUAAAUCCCGUCGGUACCCG	21	2.15	0.00	6.26	0.00	145	1.38		3
miRn-39	AGGACUGGAUCGCCGGAGGGU	21	0.00	0.00	8.12	0.00	63	0.59		1
miRn-40	AGCGGUGGAAGGGGCAUGCAGA	22	0.72	4.95	1.76	1.09	156	0.85	Yes	1
miRn-41	AGCCGUCGGACAUAAGCUUAUC	22	0.00	6.37	0.00	2.45	80	1.35		2
miRn-42	AGAGGAUUGAAGGGAUUAAAUC	22	0.00	3.23	1.37	2.00	107	1.44	Yes	2
miRn-43	AGAAACACGUCCCUGUCAGGGC	22	1.95	2.53	13.80	3.99	168	1.11		1
miRn-44	ACGACUCGAUGCUCGGCACCU	21	5.43	0.00	0.00	0.63	55	0.58		1
miRn-45	ACCGGAGGAGGUUAGAGGAGC	21	20.19	45.49	15.46	8.34	134	1.03	Yes	1
miRn-46	AAGGAGGUCCUGGACACAUAAG	22	0.00	2.93	3.82	2.09	242	1.18		1
miRn-47	AACCGGAAGACCUAGAGCUAACU	23	0.00	0.00	0.00	3.36	50	0.51		1
miRn-48	AAACAAUGUUUGGUUGCCUGGUC	23	11.07	1.72	9.00	1.18	81	0.81		3
miRn-49	AAAAGACUGAGCCGAAUUGAAAU	23	6.35	0.00	2.74	0.00	92	1.25		7

^a^ Reads are the average values of each sample after being normalized to one million with the total sequence reads of each library. ^b^ “Yes” means the existence of miRNA*. ^c^ The length means the average length of precursors of the miRNA with more than one loci. ^d^ The MFEI means the average MFEI of precursors of the miRNA with more than one loci. LP (nt), the length of precursor; MFE (kcal mol^−1^), minimal folding free energy.

## Supplementary Materials

Supplementary materials can be found at https://www.mdpi.com/1422-0067/20/13/3146/s1. Figure S1: The secondary structures of novel miRNA precursors, Table S1: The expression of known miRNAs and miRNA*s in MCMV and SCMV single- and co-infected maize libraries, Table S2: The predicted target genes of novel miRNAs, Table S3: The probes used for Northern blotting of small RNAs, Table S4: The primers used for qRT-PCR of miRNA target genes.

## Abbreviations

MCMV	maize chlorotic mottle virus
SCMV	sugarcane mosaic virus
MLN	maize lethal necrosis
miRNA	microRNA
siRNA	small interfering RNA
vsiRNA	virus-derived siRNA
qRT-PCR	quantitative real-time reverse transcription-polymerase chain reaction
