# Supplementary material for: Characterization of Maize miRNAs in Response to Synergistic Infection of Maize Chlorotic Mottle Virus and Sugarcane Mosaic Virus
Source: Int J Mol Sci. 2019 Jun 27;20(13):3146. doi: 10.3390/ijms20133146 (PMC6650953; doi:10.3390/ijms20133146)
Supplement: Supplementary file 1 [file ijms-20-03146-s001.zip › Figure S1.pdf]

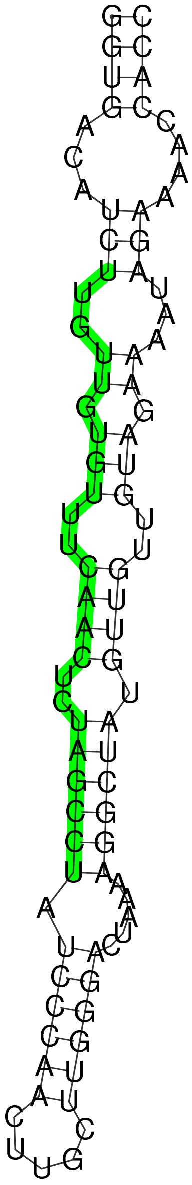

miRn-01 UUGUUGUGUUUCAACUCUAGCCU 6 140481307-140481329

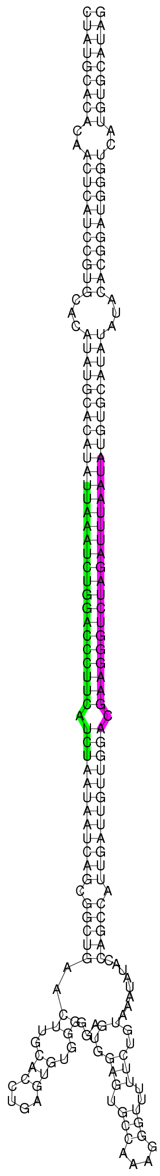

miRn-02 UUAAAUCUGGACCCUUCAUCU 6   88754543-88754563

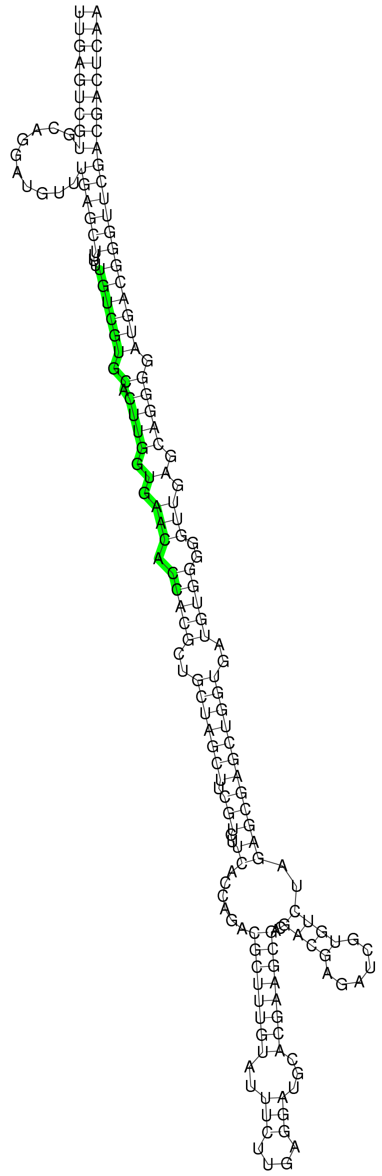

miRn-03 UGUCGUGCACUUGGUGAACACC 6 69391153-69391174

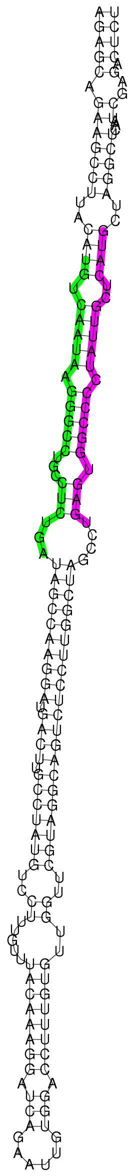

miRn-04 UGUCAAUAAGGGCCUGCCUCUGA 4 47540936-47540958

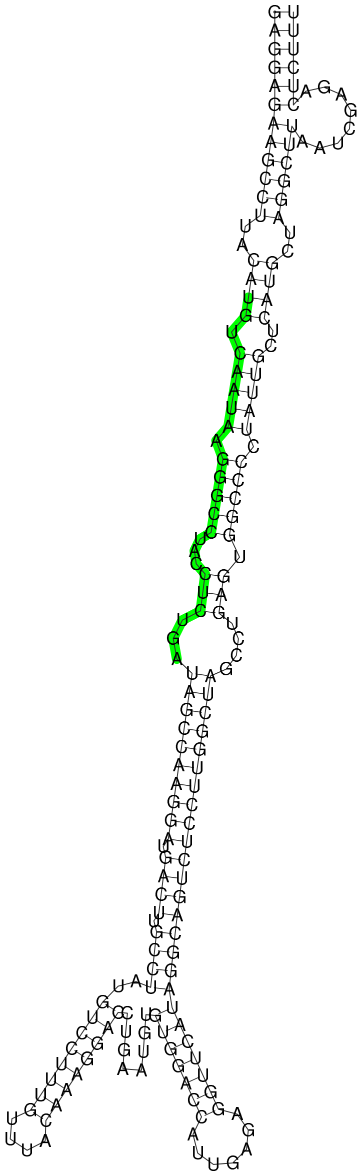

miRn-05 UGUCAAUAAGGGCCUACCUCUGA 4 47481545-47481567

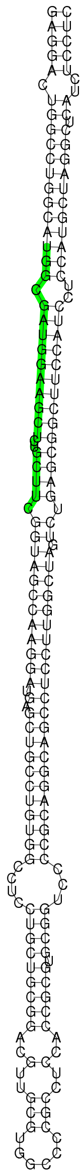

miRn-06 UGGCGAUGGAAGCUCUGCUUC   2 193350705-193350725

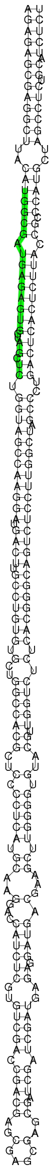

miRn-07 UGGCGAUGAGAGUGGUAGCUC 4 47269331-47269351

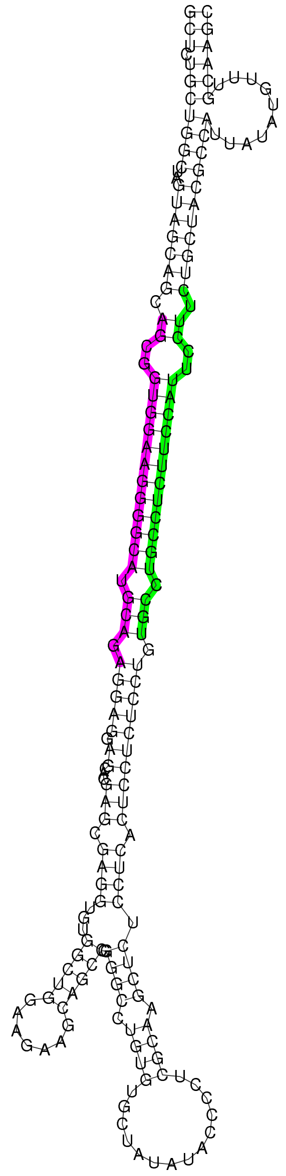

miRn-08 UGCCUGCCUCUUCCAUUCCUUC 1 6409354-6409375

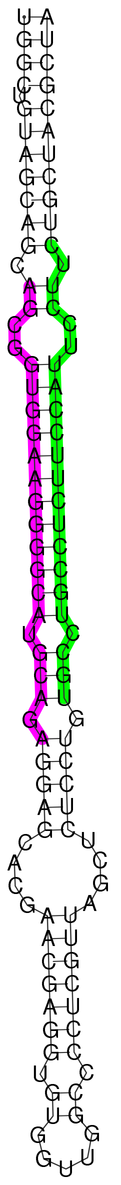

miRn-08 UGCCUGCCUCUUCCAUUCCUUC 9 153752335-153752356

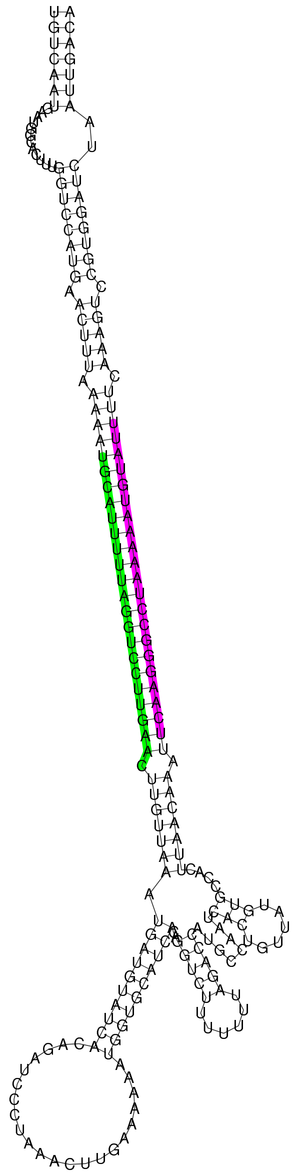

miRn-09 UGCAUUUUUAGGUCCUUGAAC 4 231951286-231951306

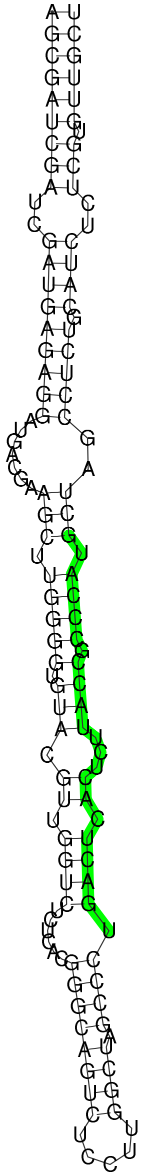

miRn-10   UGACUCACUCUUACCGCCCAUG 4 47269510-47269531

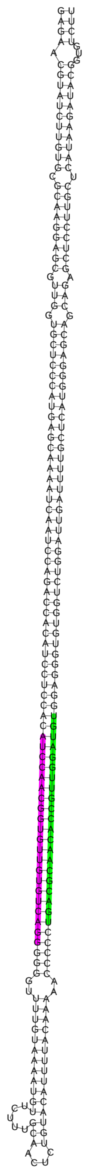

miRn-11 UGACGCAACACCGUUGGAUGU 4 225419731-225419751

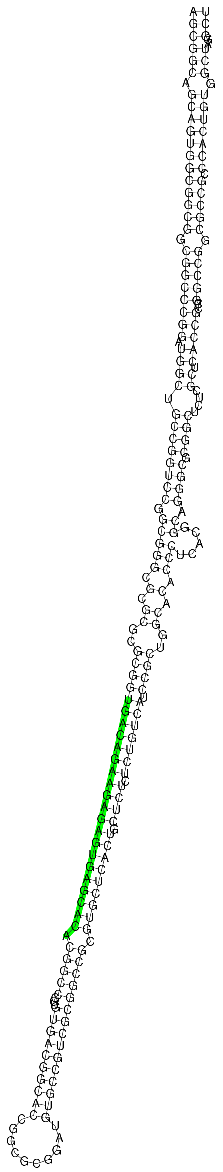

miRn-12 UGACAGAAGAGAGUGAGCACA 2 32121388-32121408

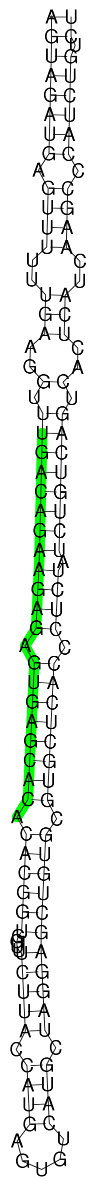

miRn-12 UGACAGAAGAGAGUGAGCACA 3 7775573-7775593

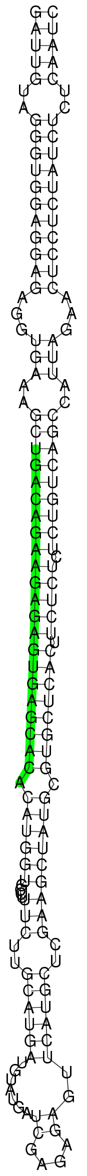

miRn-12 UGACAGAAGAGAGUGAGCACA 3 7775874-7775894

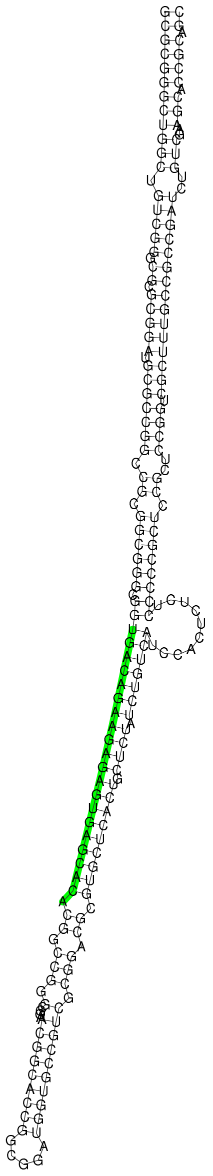

miRn-12 UGACAGAAGAGAGUGAGCACA 4 137206551-137206571

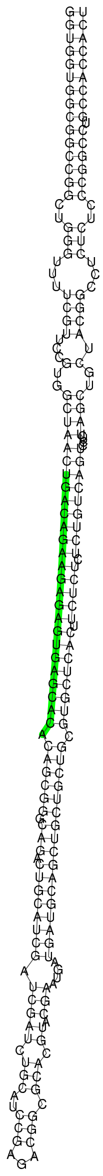

miRn-12 UGACAGAAGAGAGUGAGCACA 4 83914058-83914078

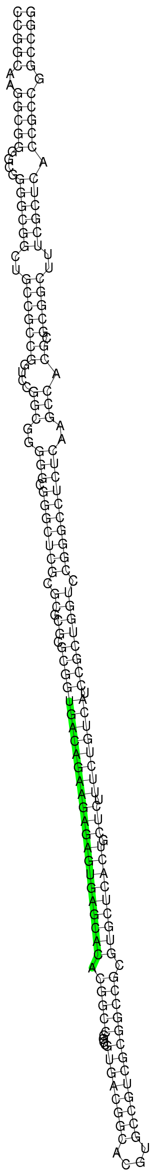

miRn-12 UGACAGAAGAGAGUGAGCACA 10 128550068-128550088

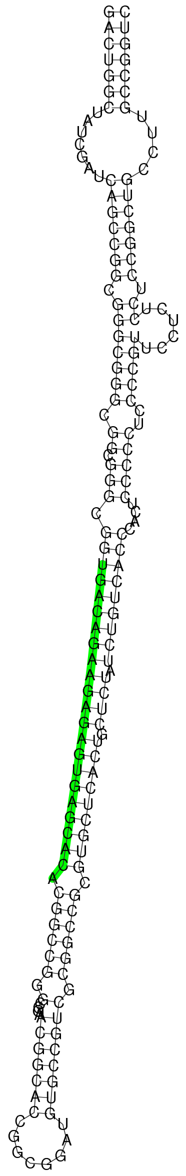

miRn-12 UGACAGAAGAGAGUGAGCACA 5 183907945-183907965

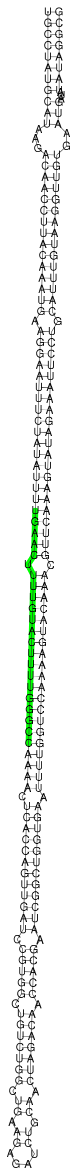

miRn-13 UGAACUUUUGUACUUUUGGGCC 10 113038375-113038396

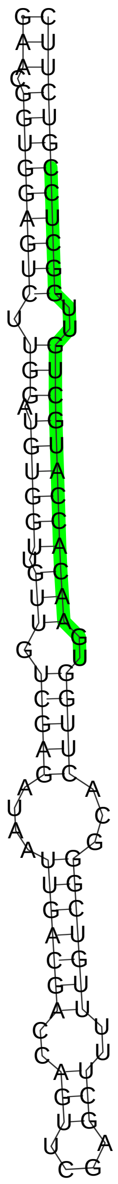

miRn-14 UGAACACCAUGCUGUUGGCUCC 7 147017886-147017907

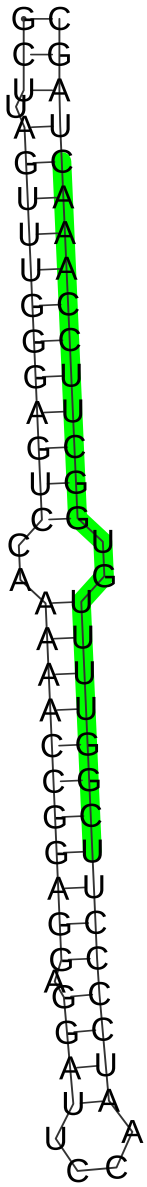

miRn-15 UCGGUUUUGUGGCUUCCAAAC 1 280086660-280086680

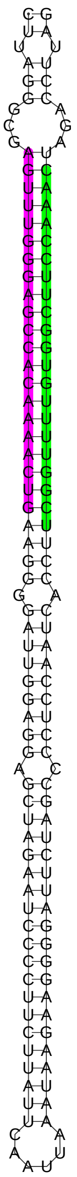

miRn-15 UCGGUUUUGUGGCUUCCAAAC 1 73390309-73390329

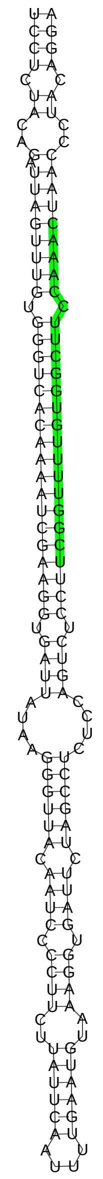

miRn-15 UCGGUUUUGUGGCUUCCAAAC 7 166772904-166772924

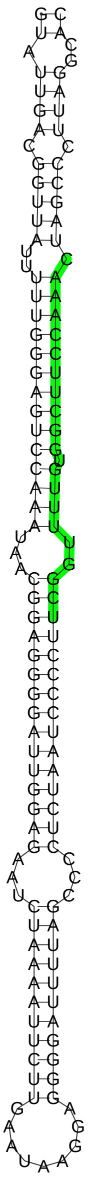

miRn-15 UCGGUUUUGUGGCUUCCAAAC 8 100779729-100779749

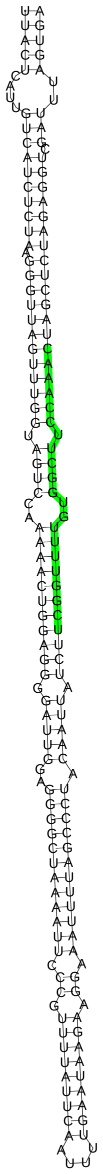

miRn-15 UCGGUUUUGUGGCUUCCAAAC 8 148688613-148688633

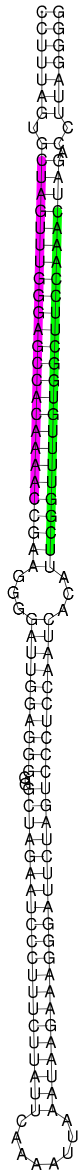

miRn-15 UCGGUUUUGUGGCUUCCAAAC 5 214383005-214383025

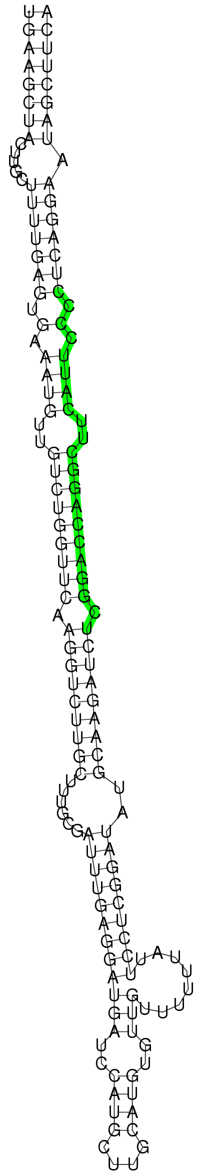

miRn-16 UCGGACCAGGCUUCAUUCCCC 1 223371679-223371699

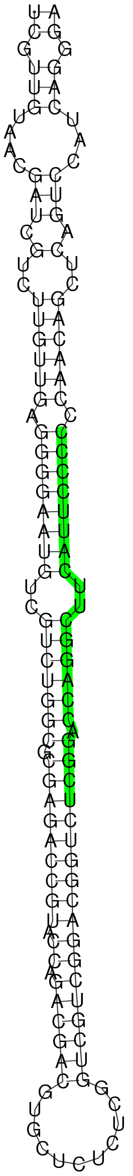

miRn-16 UCGGACCAGGCUUCAUUCCCC 1 241357125-241357145

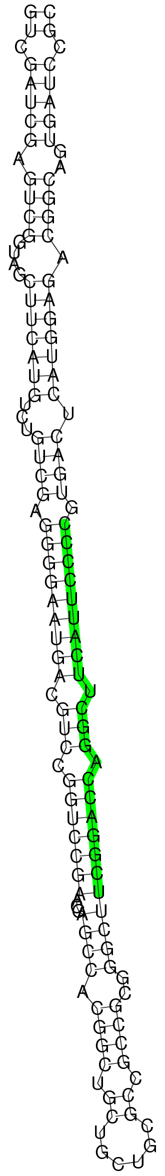

miRn-16 UCGGACCAGGCUUCAUUCCCC 1 274783711-274783731

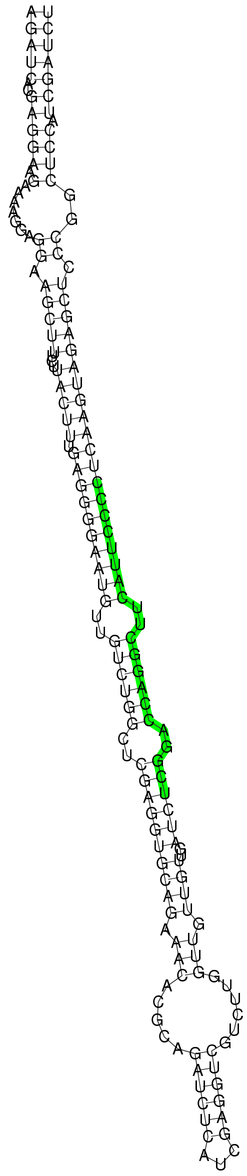

miRn-16 UCGGACCAGGCUUCAUUCCCC 4 150433956-150433976

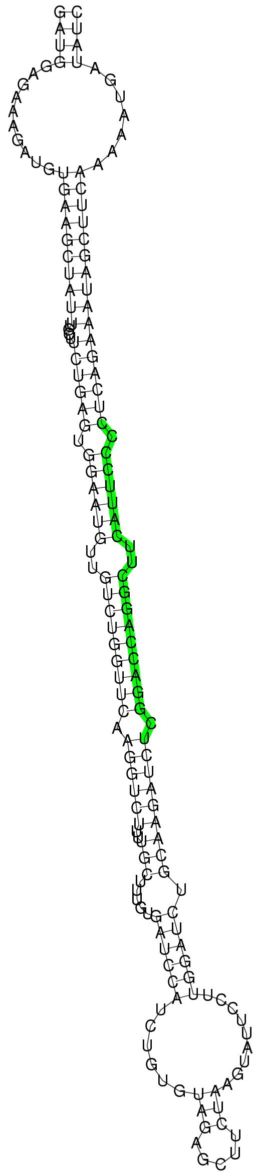

miRn-16 UCGGACCAGGCUUCAUUCCCC 5 21954975-21954995

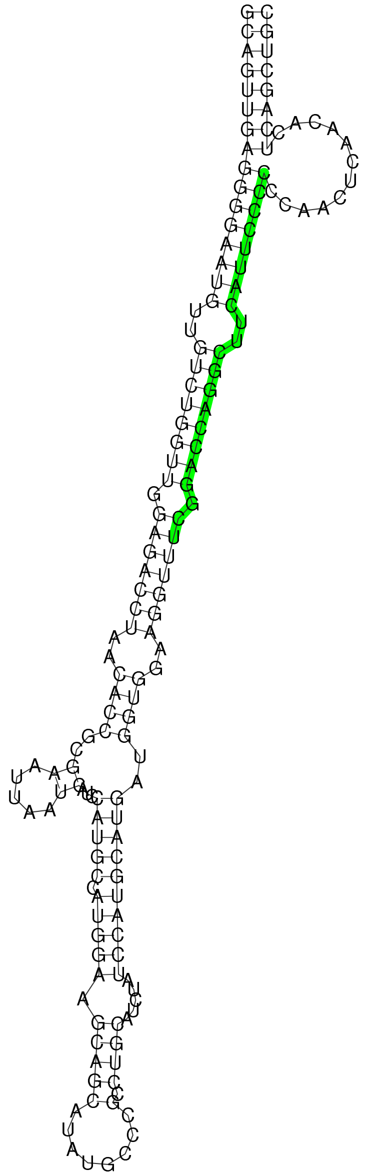

miRn-16 UCGGACCAGGCUUCAUUCCCC 1 13558900-13558920

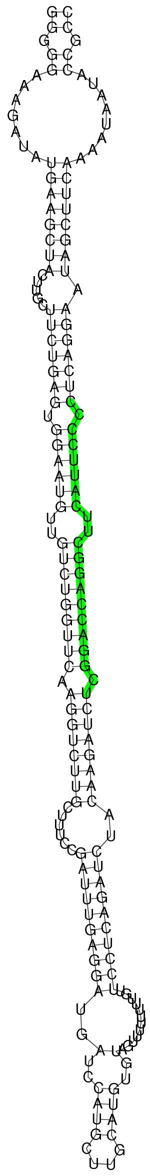

miRn-16 UCGGACCAGGCUUCAUUCCCC 1 223279185-223279205

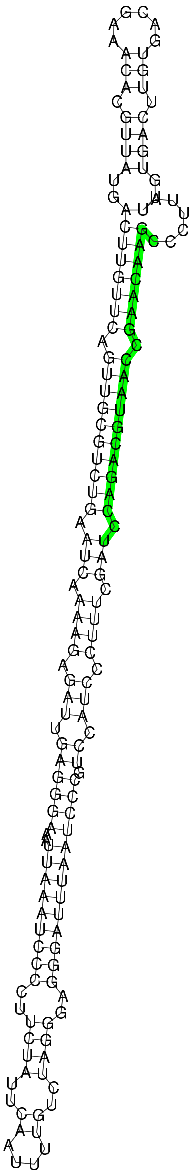

miRn-17 UCCAGACGUAACCGAACAAGC 1 103180104-103180124

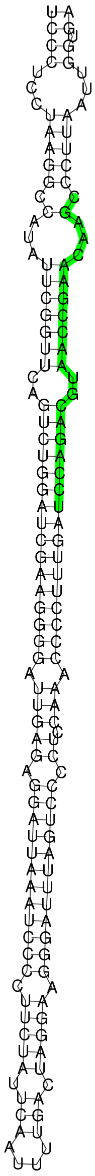

miRn-17 UCCAGACGUAACCGAACAAGC 1 58367744-58367764

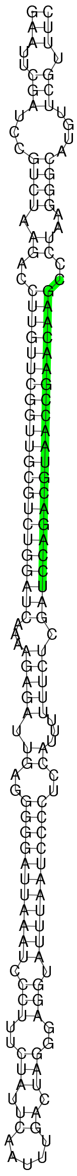

miRn-17 UCCAGACGUAACCGAACAAGC 2 103558501-103558521

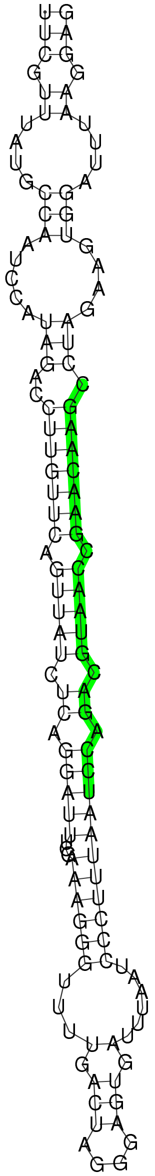

miRn-17 UCCAGACGUAACCGAACAAGC 2 207618715-207618735

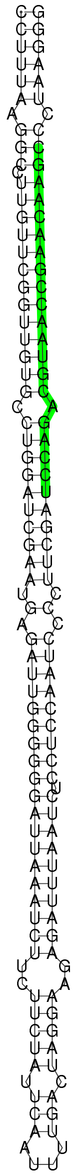

miRn-17 UCCAGACGUAACCGAACAAGC 3 212520432 212520452

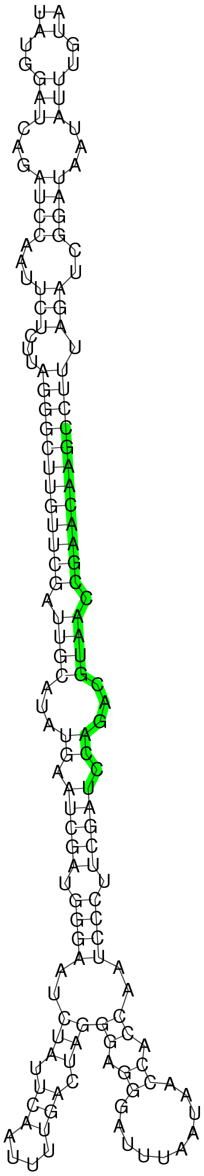

miRn-17 UCCAGACGUAACCGAACAAGC 3 45423738-45423758

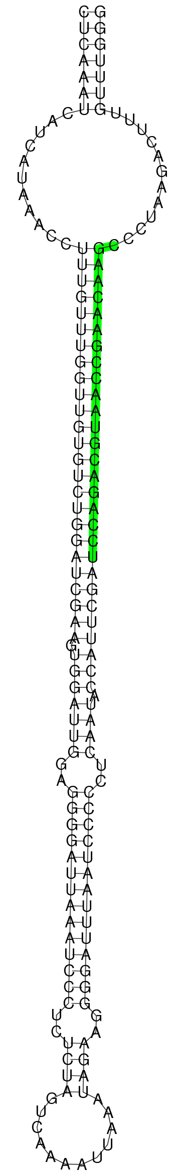

miRn-17 UCCAGACGUAACCGAACAAGC 4 236152592-236152612

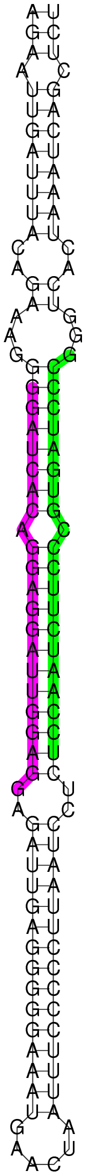

miRn-18 UCCAAUCUUCCCGUGAUCCCG 10 143953657-143953677

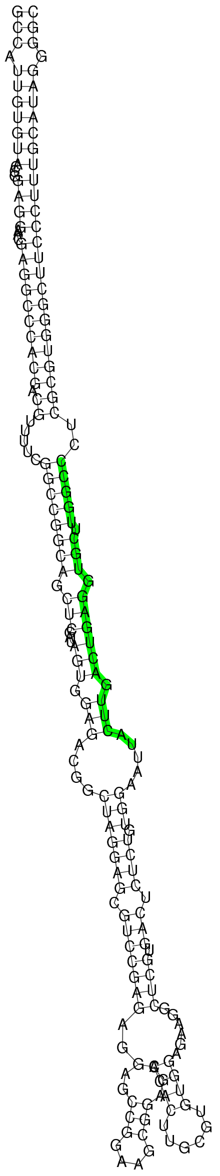

miRn-19 UACUUGACUGAGGUGCUUGGCC 1 37952896-37952917

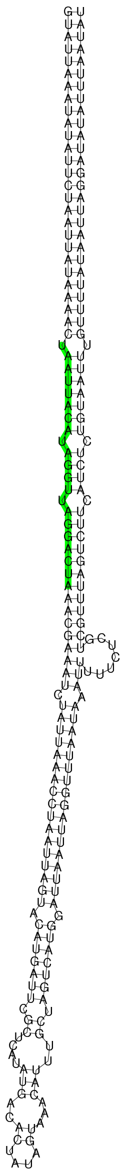

miRn-20 UAAUUACAUAGGUUAGGACUA 1 124697955-124697975

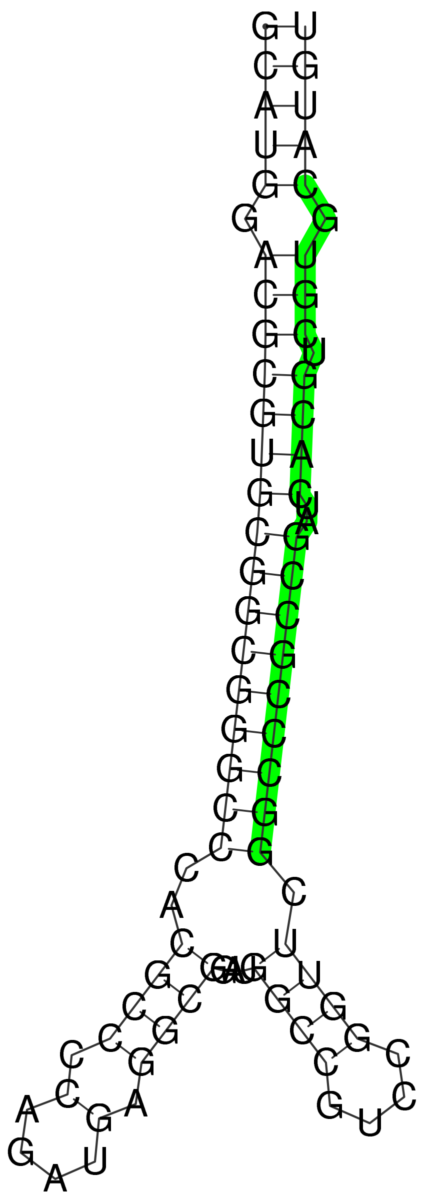

miRn-21 GGCCCGCCGAUCACGUCGUGC 5 107506390-107506410

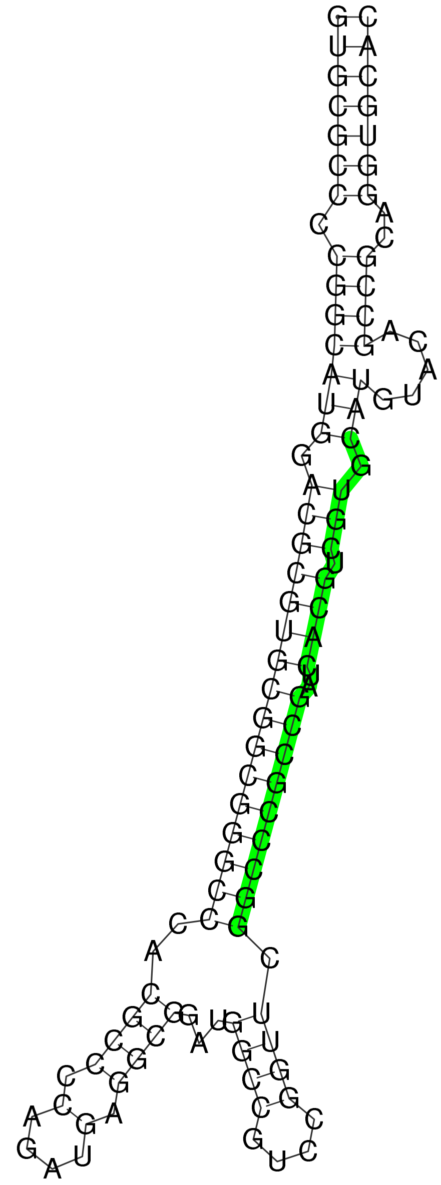

miRn-21 GGCCCGCCGAUCACGUCGUGC 7 1271358-1271378

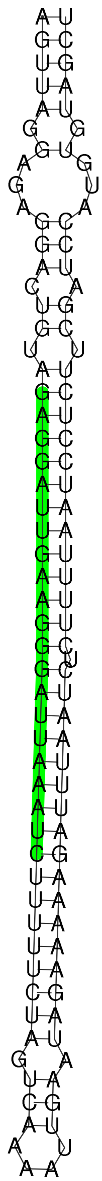

miRn-22 GAGGAUUGAAGGGAUUAAAUC 4 191157907-191157927

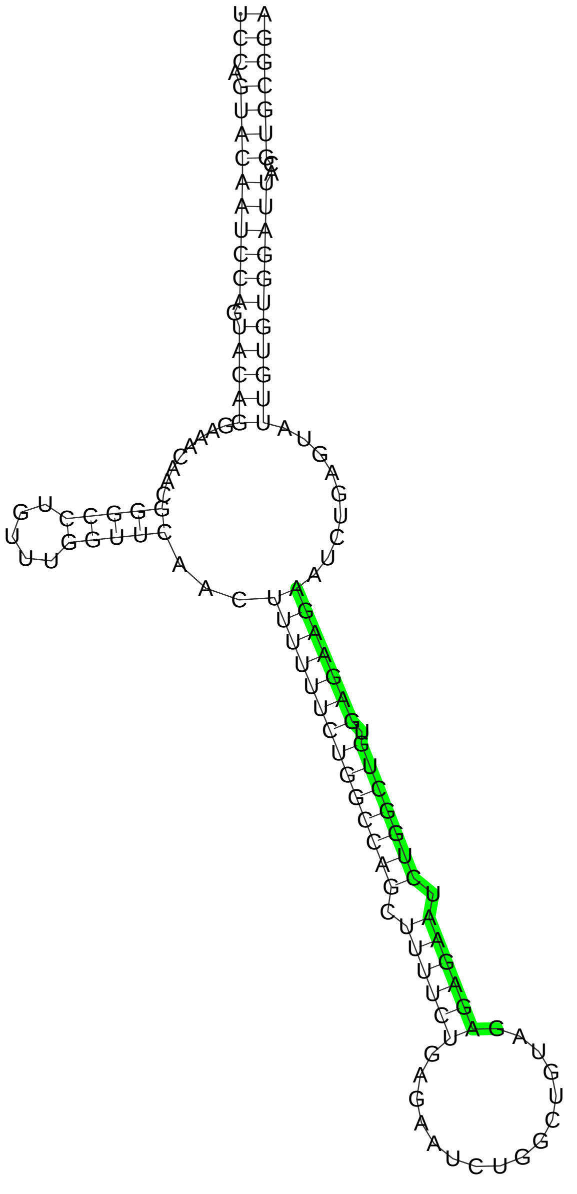

miRn-23 GAGAGAAUCUGGCUGUGAGAAGA 3 221435748-221435770

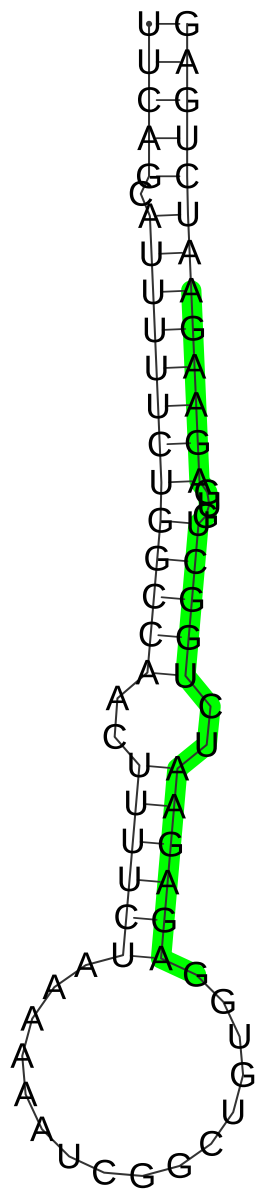

miRn-23 GAGAGAAUCUGGCUGUGAGAAGA 10 57751381-57751403

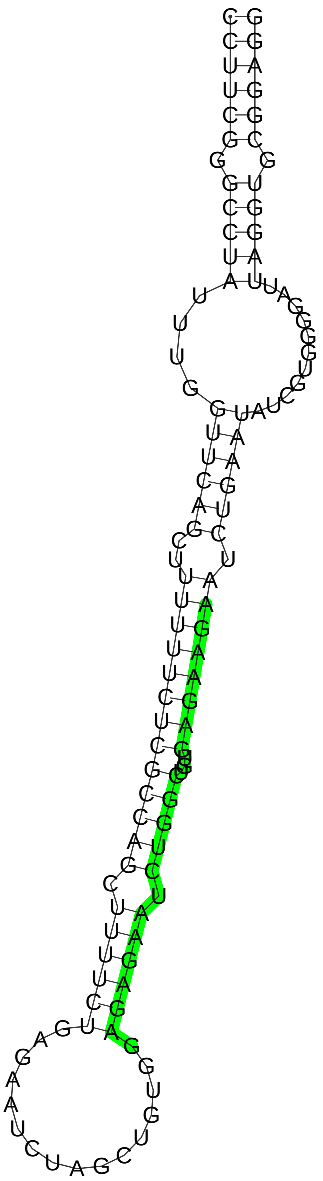

miRn-23 GAGAGAAUCUGGCUGUGAGAAGA 2 138272974-138272996

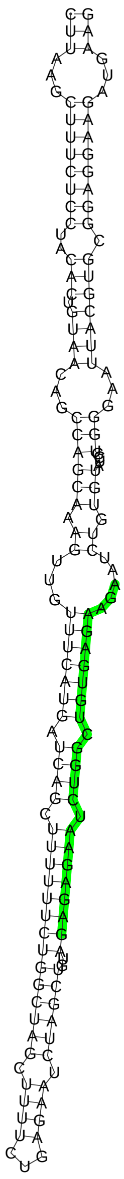

miRn-23 GAGAGAAUCUGGCUGUGAGAAGA 2 30501798-30501820

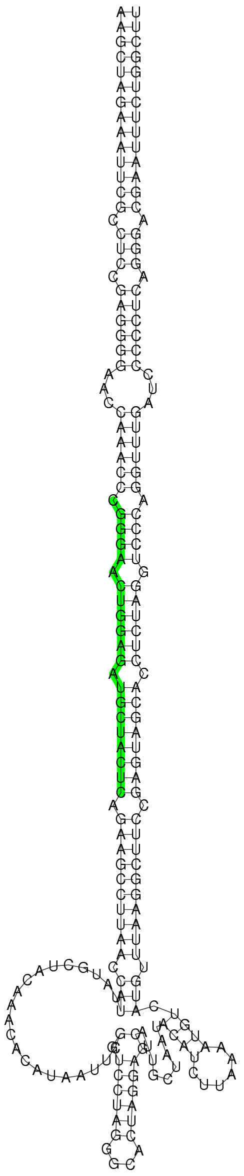

miRn-24 CGGGAACUGGAGAUGCUACUC 5 43837142-43837162

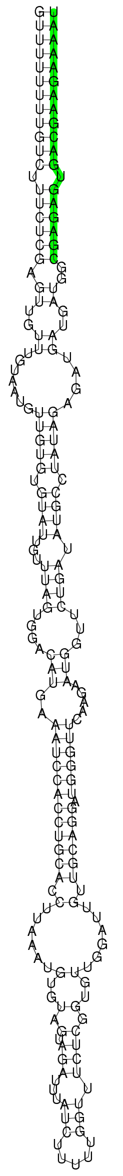

miRn-25 CGAGAGUGACGAAGAAAAUCGA 1 153585144-153585165

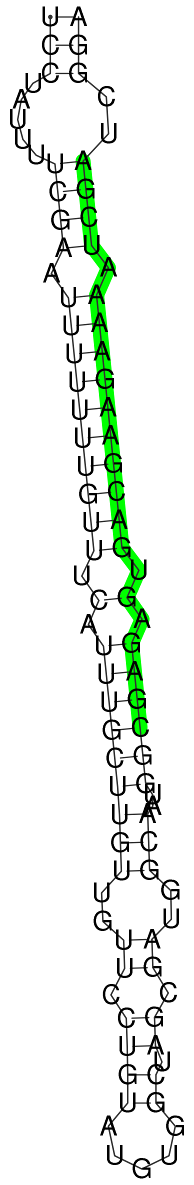

miRn-25 CGAGAGUGACGAAGAAAAUCGA 7 8223605-8223626

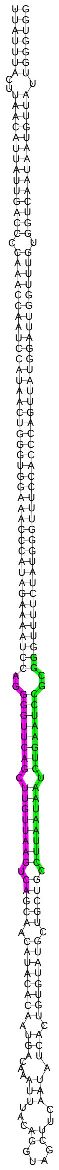

miRn-26 CCUUAAUAAUCUGAAUCCGCGG 3 187225200-187225221

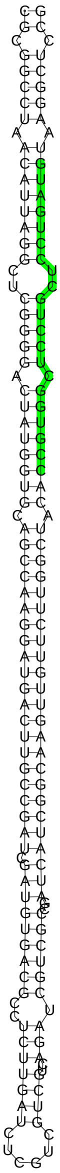

miRn-27 CCGUGGCUCCUGCUCCUGAUG 3 37610571-37610591

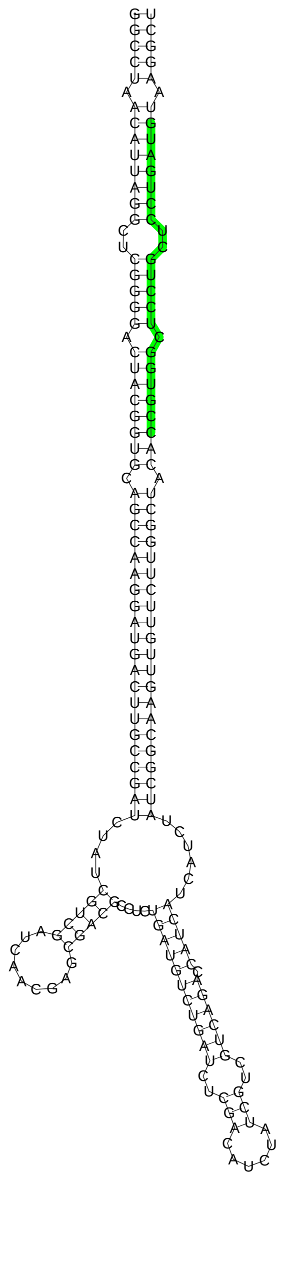

miRn-27 CCGUGGCUCCUGCUCCUGAUG 8 4792853-4792873

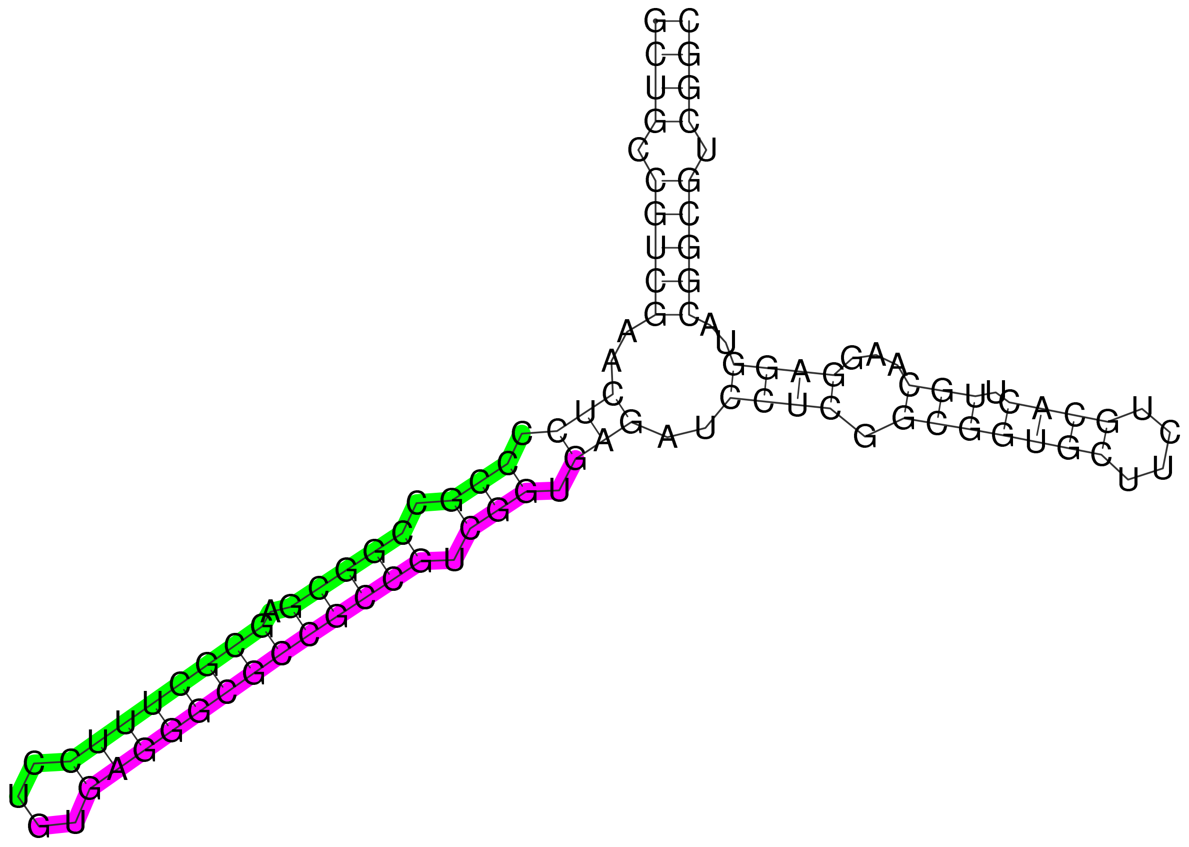

miRn-28 CCCGCCGGCGAGCGCUUUCCU 1 116730726-116730746

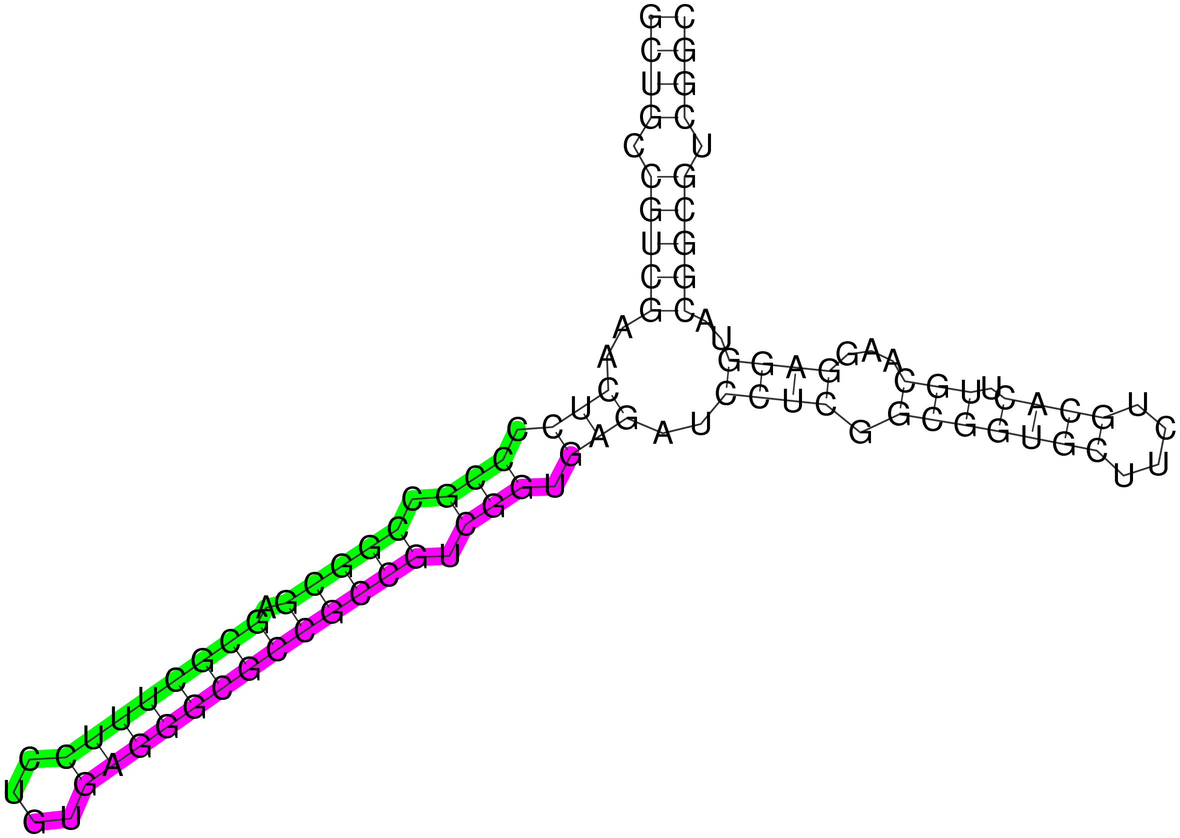

miRn-28 CCCGCCGGCGAGCGCUUUCCU 1 116752622-116752642

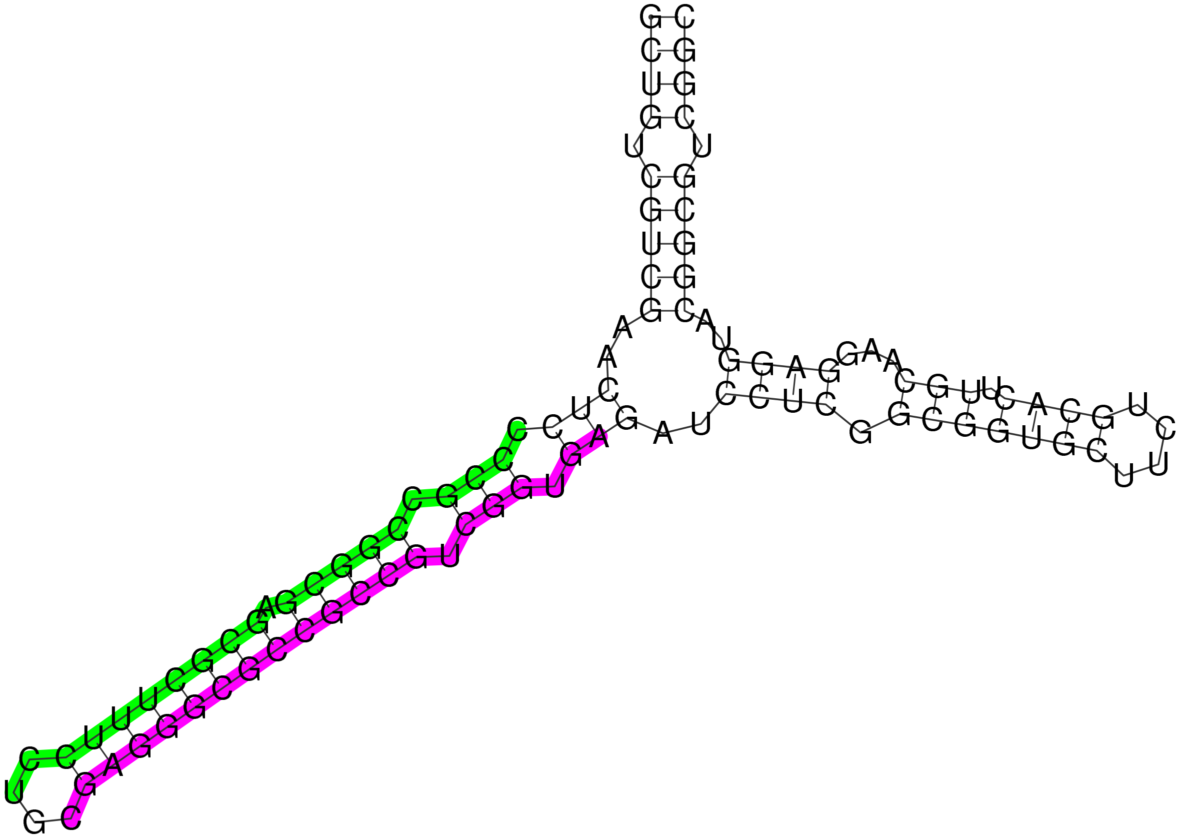

miRn-28 CCCGCCGGCGAGCGCUUUCCU 6 33172057-33172077

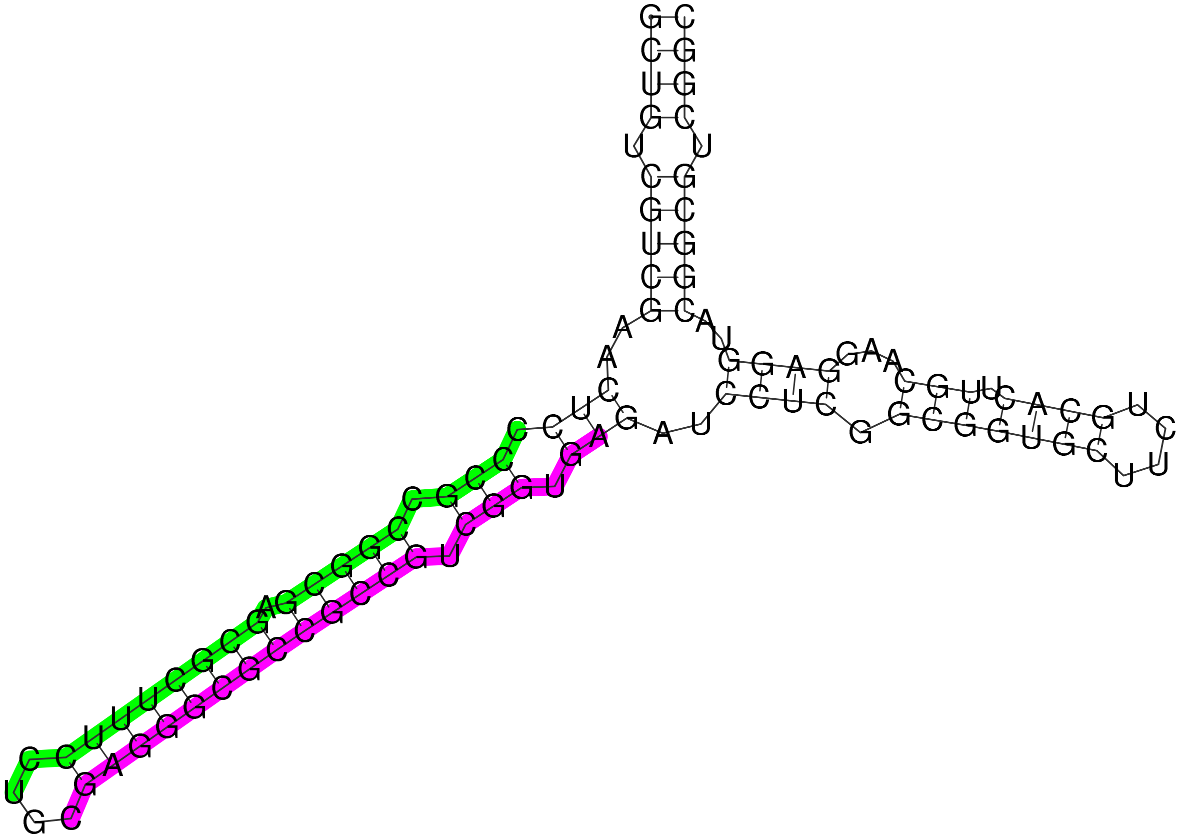

miRn-28 CCCGCCGGCGAGCGCUUUCCU   6 33433221-33433241

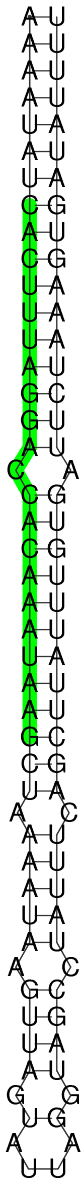

miRn-29 CACUUUAGGACCACAAAUAAG 2 47194756-47194776

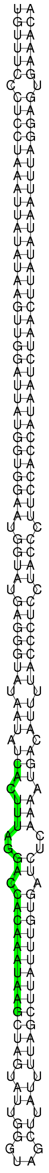

miRn-29 CACUUUAGGACCACAAAUAAG 6 139139095-139139115

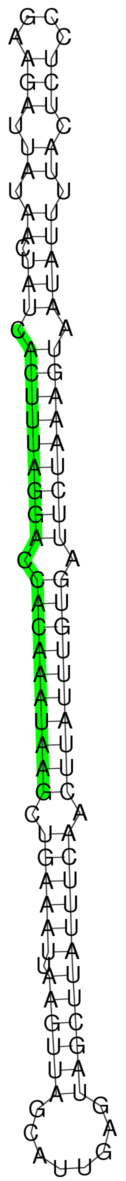

miRn-29 CACUUUAGGACCACAAAUAAG 7 38554855-38554875

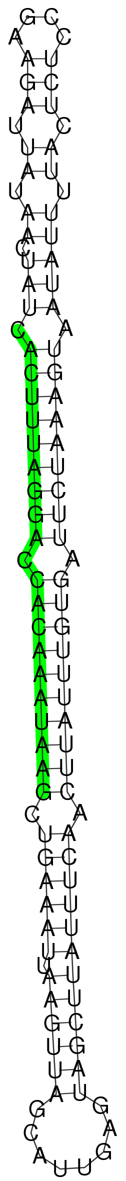

miRn-29 CACUUUAGGACCACAAAUAAG 7   38566694-38566714

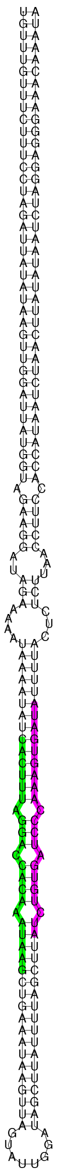

miRn-29 CACUUUAGGACCACAAAUAAG 8 96988193-96988213

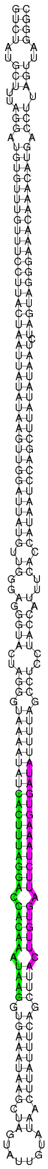

miRn-29 CACUUUAGGACCACAAAUAAG 9 138750554-138750574

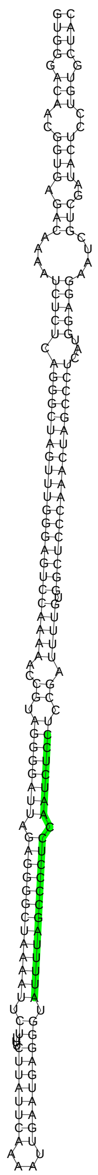

miRn-34 AUUUUAGCCCCUCCAAUCUCC 10 64849934-64849954

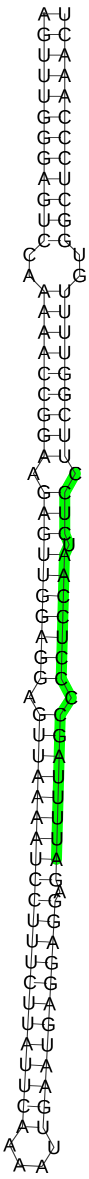

miRn-34 AUUUUAGCCCCUCCAAUCUCC 1 31812905-31812925

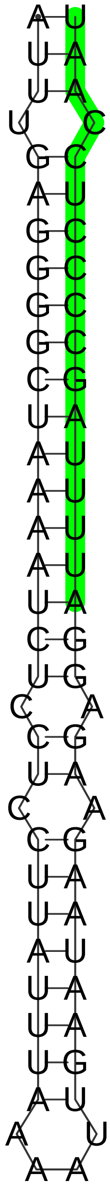

miRn-34 AUUUUAGCCCCUCCAAUCUCC 1 69703149-69703169

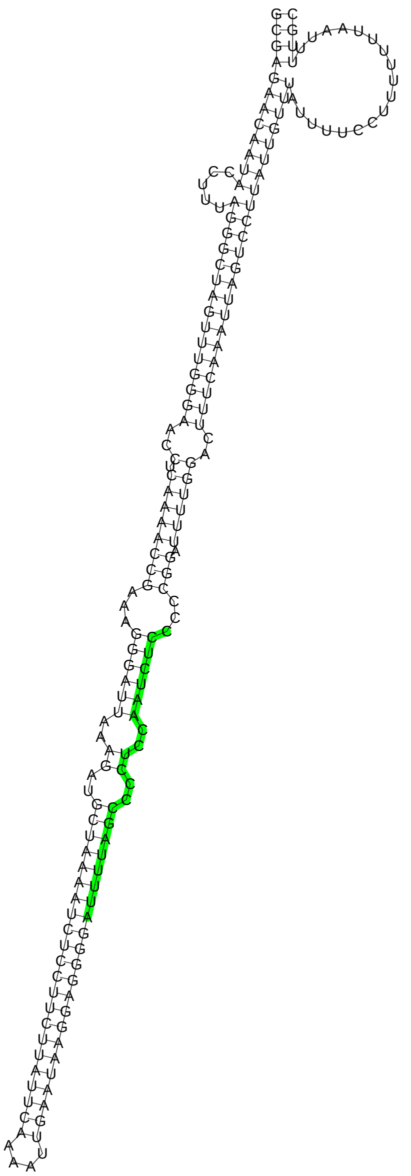

miRn-34 AUUUUAGCCCCUCCAAUCUCC 2 210616137-210616157

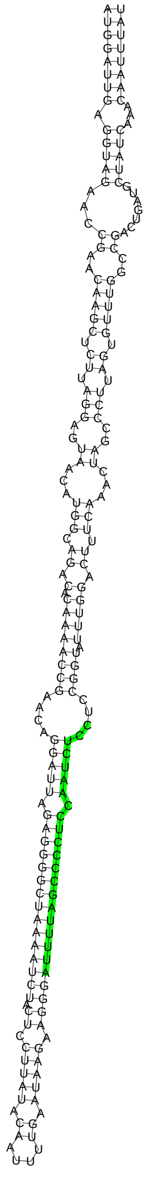

miRn-34 AUUUUAGCCCCUCCAAUCUCC 2 88590856-88590876

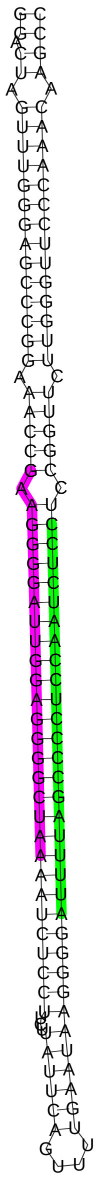

miRn-34 AUUUUAGCCCCUCCAAUCUCC 5 149512578-149512598

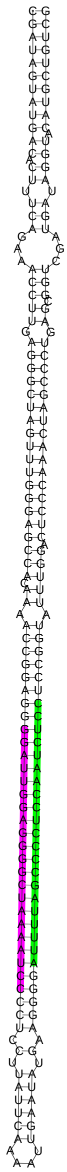

miRn-34 AUUUUAGCCCCUCCAAUCUCC 5 16452169-16452189

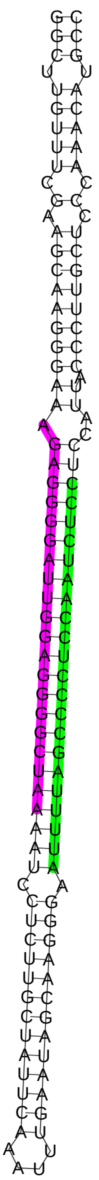

miRn-34 AUUUUAGCCCCUCCAAUCUCC 5 205836980-205837000

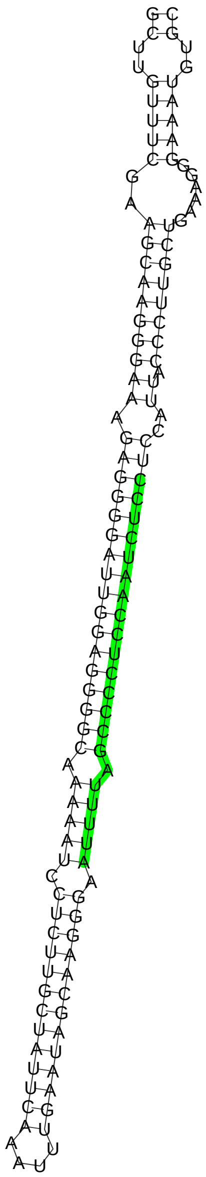

miRn-34 AUUUUAGCCCCUCCAAUCUCC 5 62942685-62942705

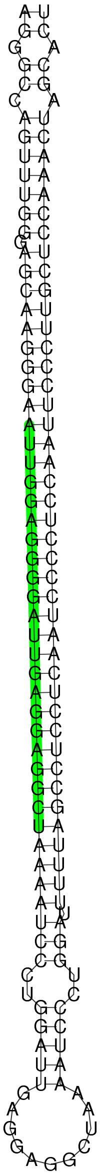

miRn-35 AUUGGAGGGGAUUGAGGAGGCU 2 78410414-78410435

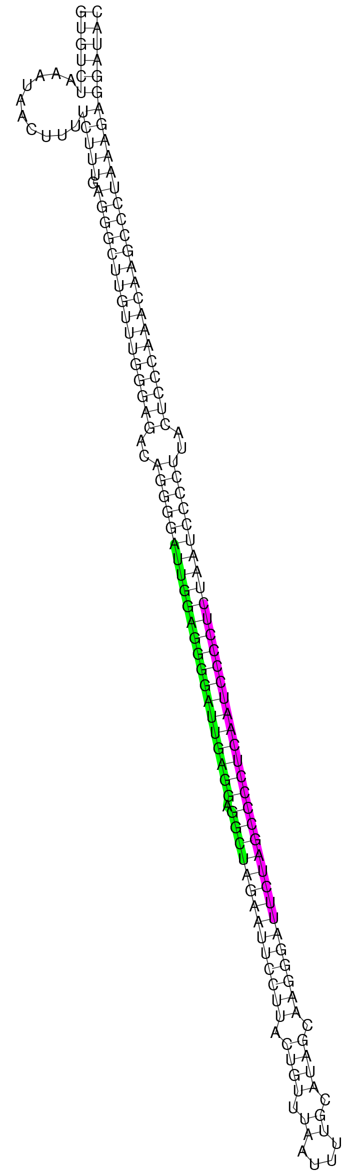

miRn-35 AUUGGAGGGGAUUGAGGAGGCU 3 179470162-179470183

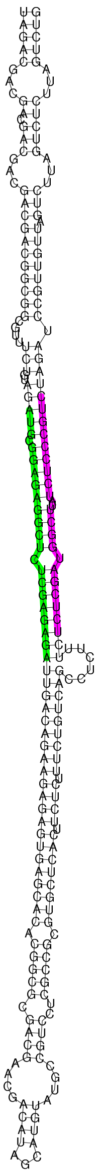

miRn-36 AUGCGGAGAGGCUCUCGAGAGA 5 92406338-92406359

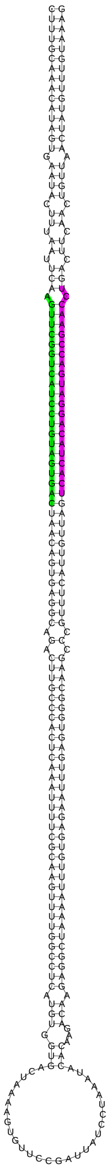

miRn-37 AGUUCGGUCAUCCUGUAGUGAC 1 192318649-192318670

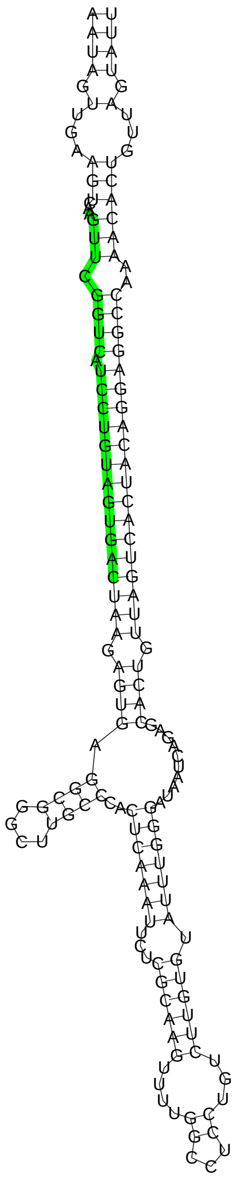

miRn-37 AGUUCGGUCAUCCUGUAGUGAC 2 219485057-219485078

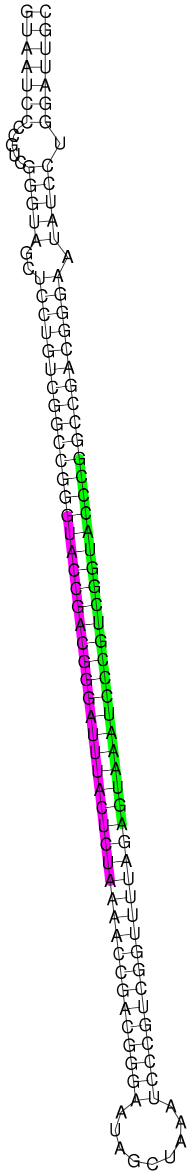

miRn-38 AGUAAAUCCCGUCGGUACCCG 1 12338494-12338514

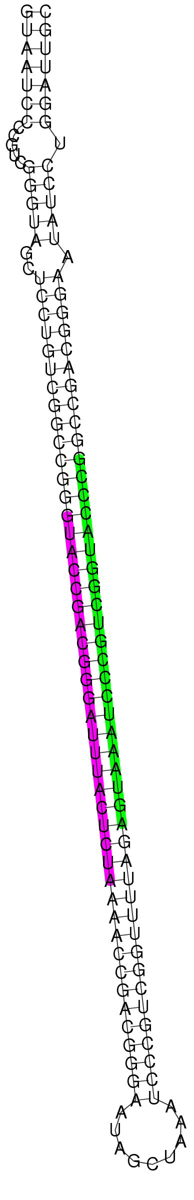

miRn-38 AGUAAAUCCCGUCGGUACCCG 2 60687852-60687872

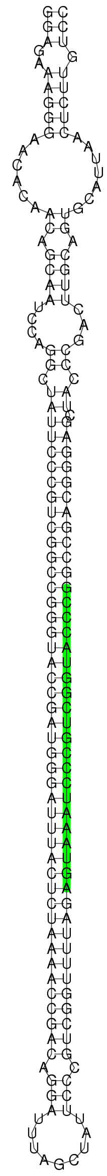

miRn-38 AGUAAAUCCCGUCGGUACCCG 3 117399050-117399070

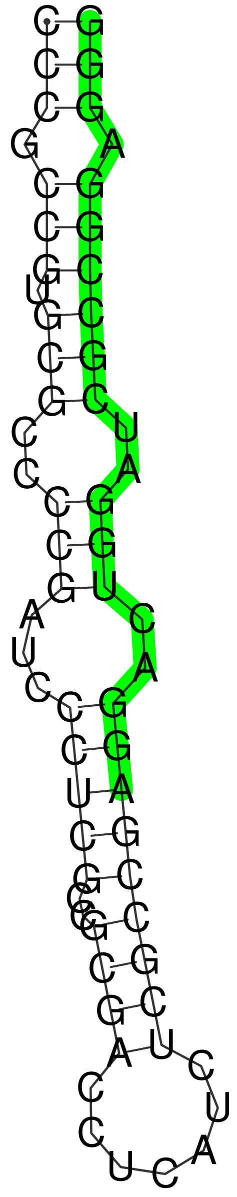

miRn-39 AGGACUGGAUCGCCGGAGGGU 10 147970442-147970462

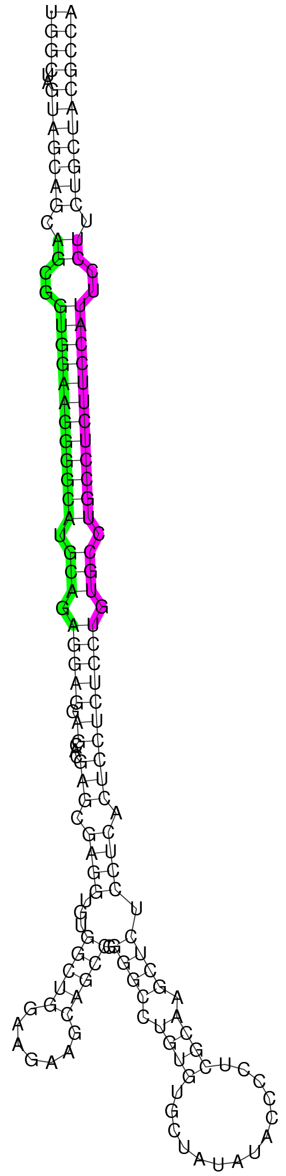

miRn-40 AGCGGUGGAAGGGGCAUGCAGA 1 6409244-6409265

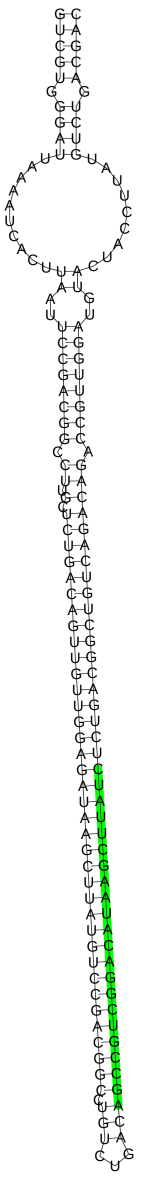

miRn-41 AGCCGUCGGACAUAAGCUUAUC 2 225192107-225192128

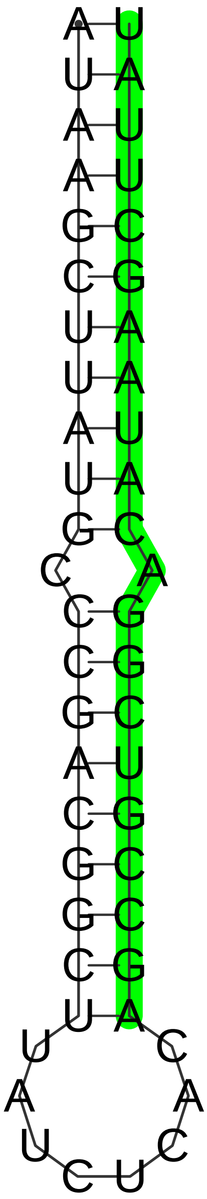

miRn-41 AGCCGUCGGACAUAAGCUUAUC 4 10816393-10816414

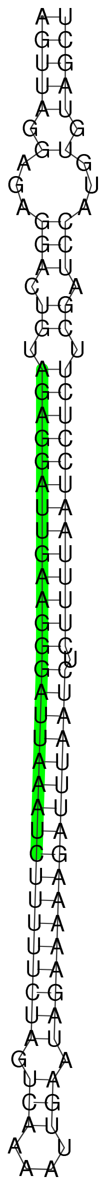

miRn-42 AGAGGAUUGAAGGGAUUAAAUC 4 191157907-191157928

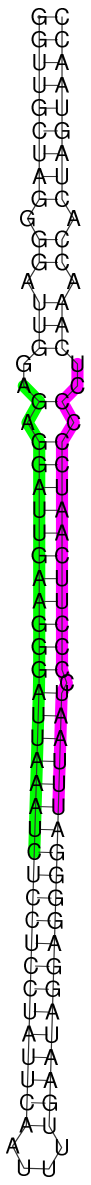

miRn-42 AGAGGAUUGAAGGGAUUAAAUC 4 231272043-231272064

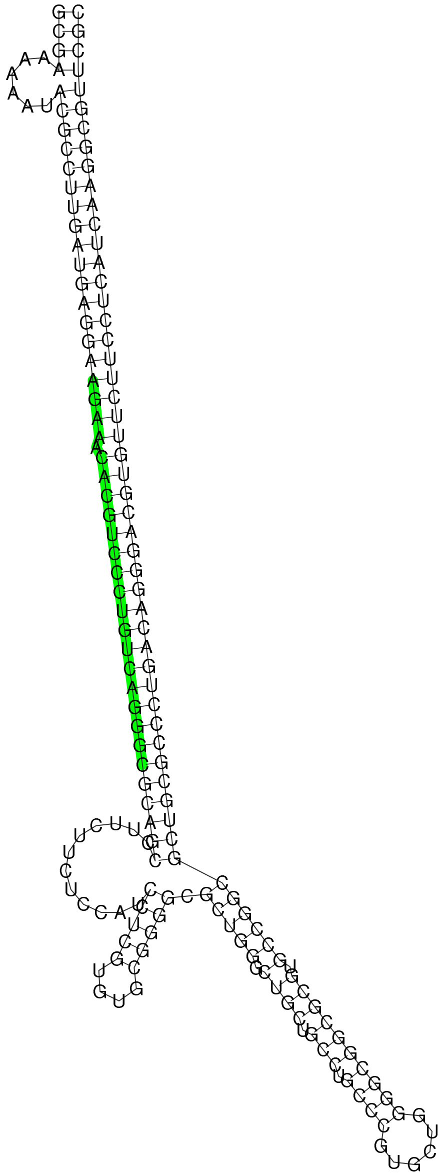

miRn-43 AGAAACACGUCCCUGUCAGGGC 7 21650640-21650661

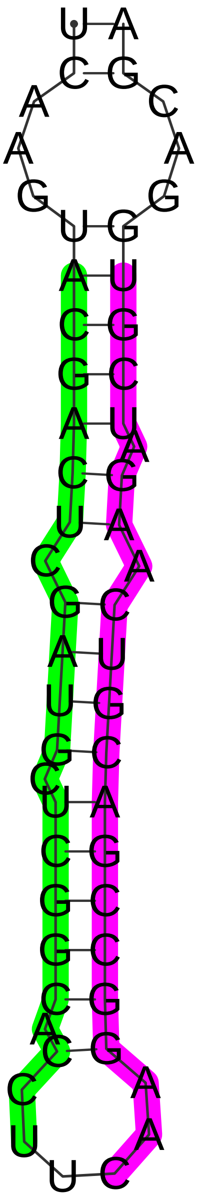

miRn-44 ACGACUCGAUGCUCGGCACCU 1 6405213-6405233

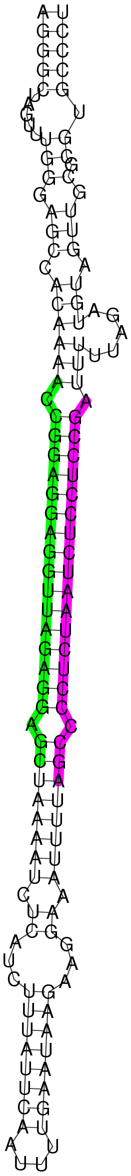

miRn-45 ACCGGAGGAGGUUAGAGGAGC 4 195967351-195967371

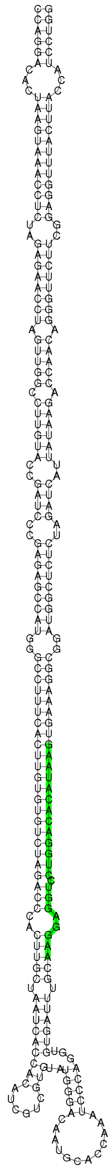

miRn-46 AAGGAGGUCCUGGACACAUAAG 7 161871296-161871317

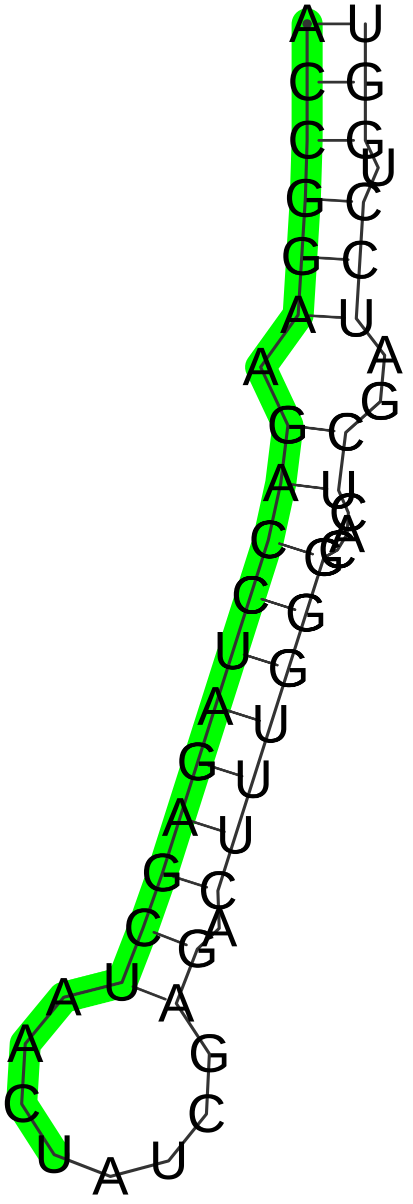

miRn-47 AACCGGAAGACCUAGAGCUAACU 7 150450558-150450580

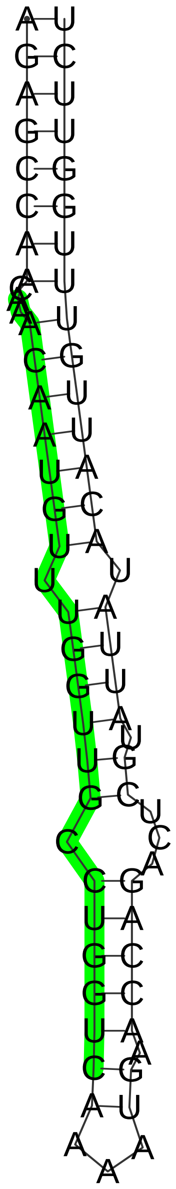

miRn-48 AAACAAUGUUUGGUUGCCUGGUC 2 206221452-206221474

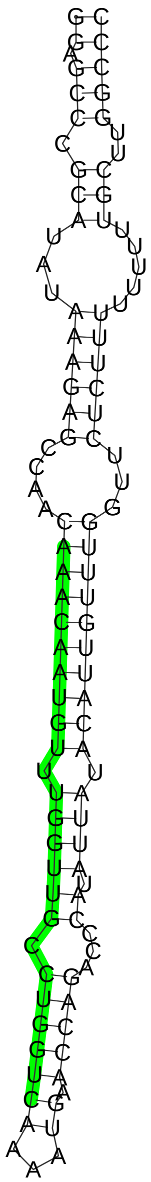

miRn-48 AAACAAUGUUUGGUUGCCUGGUC 8 101383955-101383977

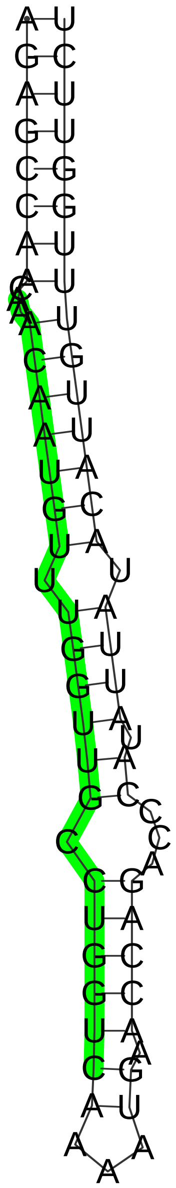

miRn-48 AAACAAUGUUUGGUUGCCUGGUC 9 22055193-22055215

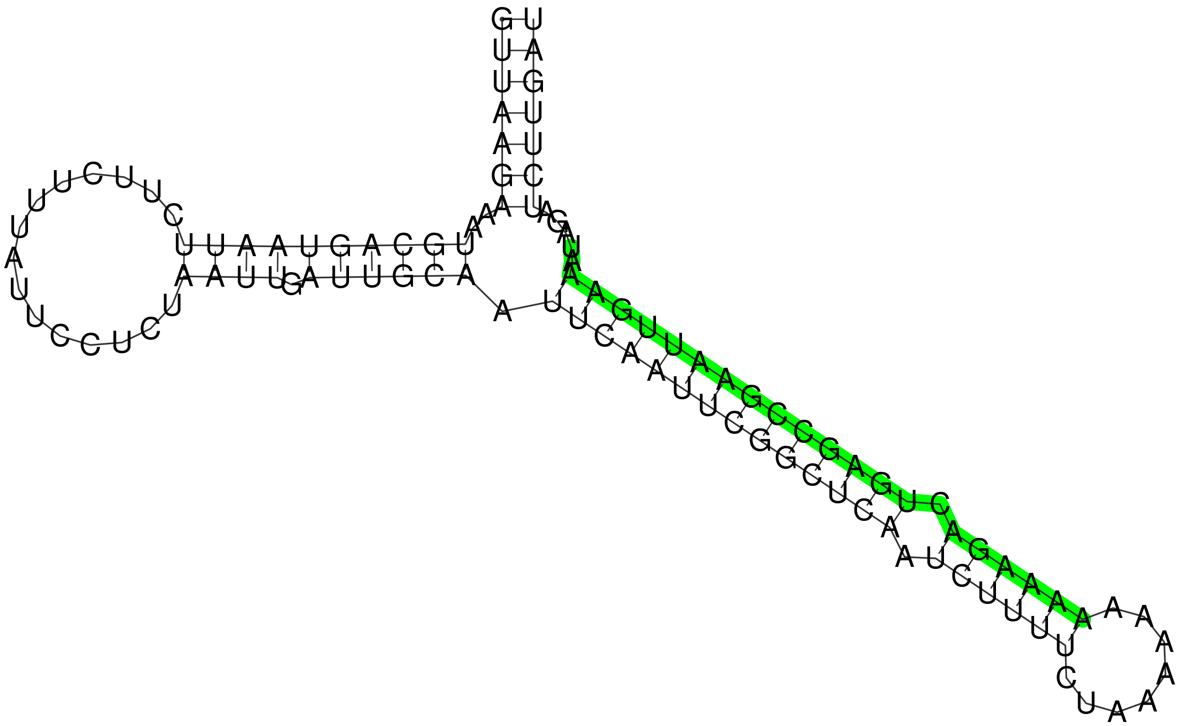

miRn-49 AAAAGACUGAGCCGAAUUGAAAU 1 203965714-203965736

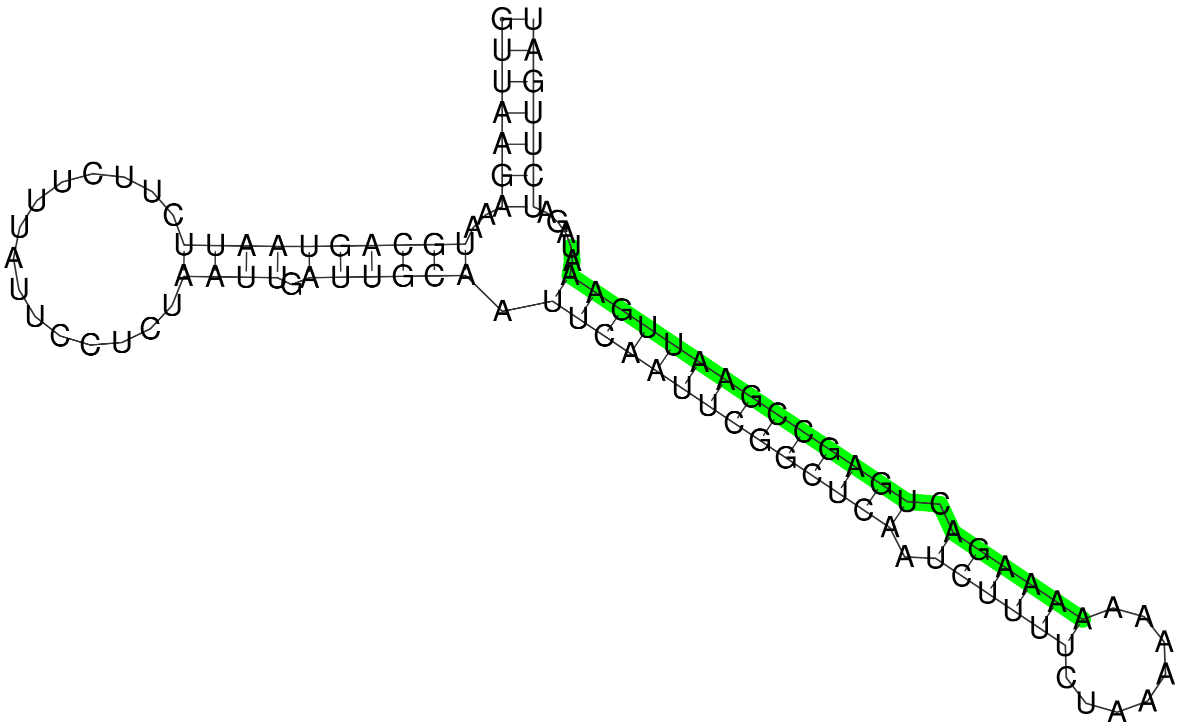

miRn-49 AAAAGACUGAGCCGAAUUGAAAU 1 234717275-234717297

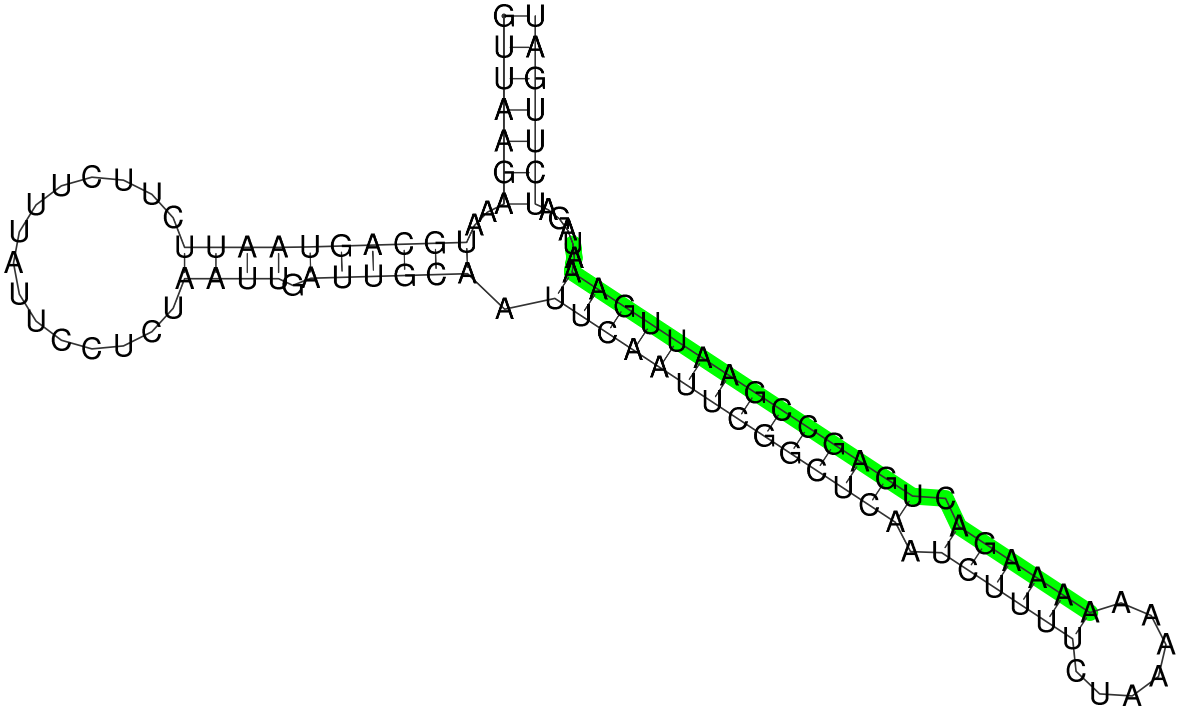

miRn-49 AAAAGACUGAGCCGAAUUGAAAU 2 200754971-200754993

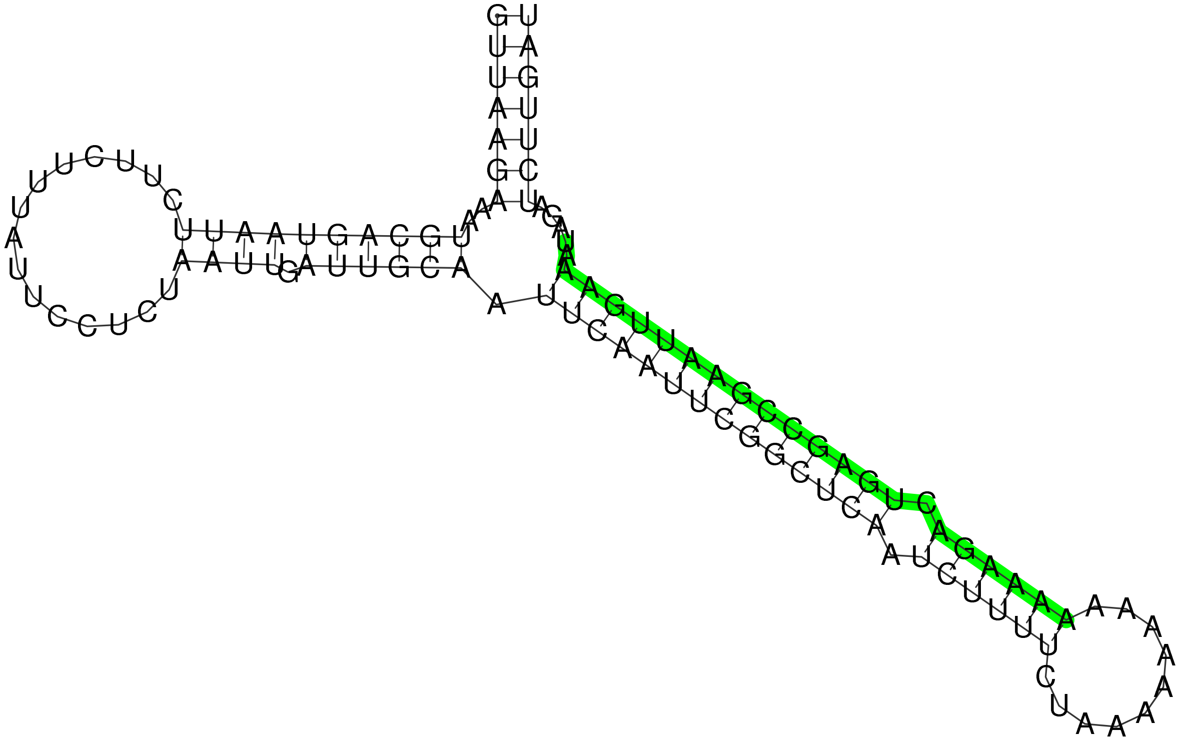

miRn-49 AAAAGACUGAGCCGAAUUGAAAU 6 160981459-160981481

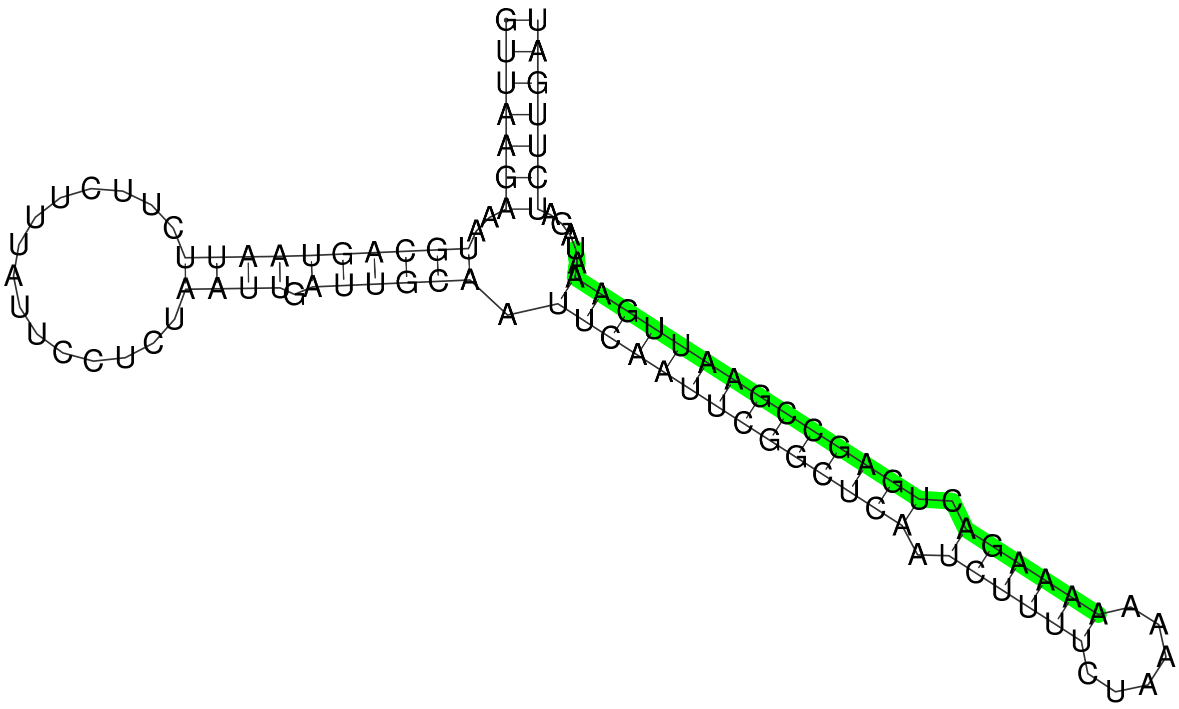

miRn-49 AAAAGACUGAGCCGAAUUGAAAU 9 33384532-33384554

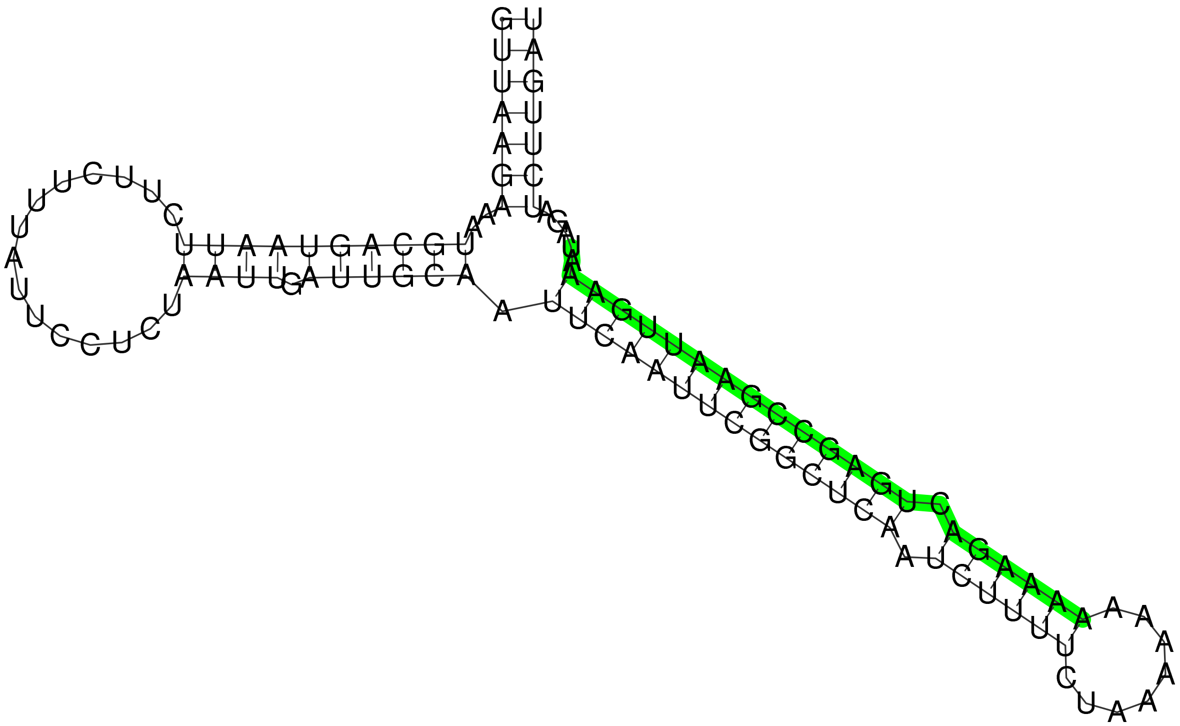

miRn-49 AAAAGACUGAGCCGAAUUGAAAU Mt 348891-348913

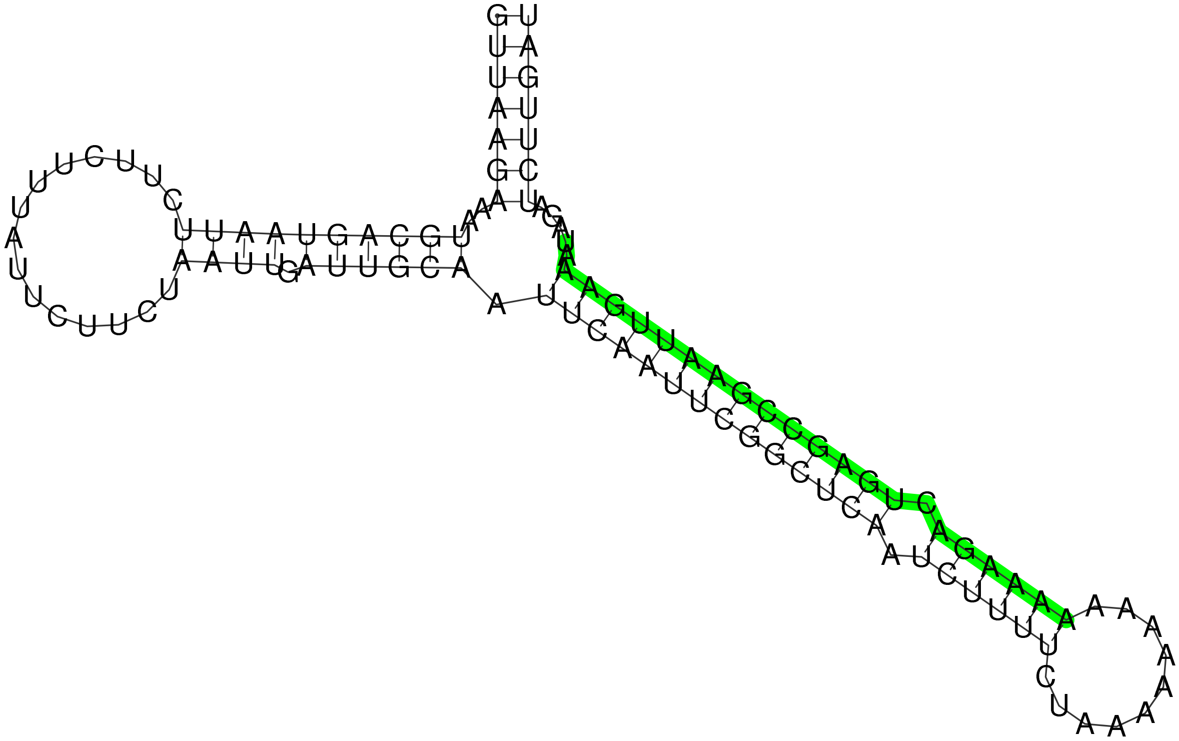

miRn-49 AAAAGACUGAGCCGAAUUGAAAU Pt 58419-58441
